# Association between serum glucose potassium ratio and short- and long-term all-cause mortality in patients with sepsis admitted to the intensive care unit: a retrospective analysis based on the MIMIC-IV database

**DOI:** 10.3389/fendo.2025.1555082

**Published:** 2025-07-30

**Authors:** Jiaqi Lou, Ziyi Xiang, Xiaoyu Zhu, Jingyao Song, Shengyong Cui, Jiliang Li, Guoying Jin, Neng Huang, Youfen Fan, Sida Xu

**Affiliations:** ^1^ Burn Department, Ningbo No. 2 Hospital, Ningbo, Zhejiang, China; ^2^ Institute of Pathology, Faculty of Medicine, University of Bonn, Bonn, Germany; ^3^ Health Science Center, Ningbo University, Ningbo, Zhejiang, China; ^4^ School of Mental Health, Wenzhou Medical University, Wenzhou, Zhejiang, China

**Keywords:** intensive care unit, MIMIC, mortality, sepsis, glucose potassium ratio, long term, Cox regression

## Abstract

**Background:**

The glucose potassium ratio (GPR) is emerging as a biomarker for predicting clinical outcomes in various conditions. However, its value in sepsis patients admitted to the intensive care unit (ICU) remains unclear. Prior studies have shown conflicting results, with some indicating GPR’s potential as an early warning indicator of metabolic decompensation in septic patients, while others found no significant association. The current study addresses these inconsistencies by conducting the first large-scale, systematic validation of GPR in ICU sepsis patients.

**Methods:**

This retrospective cohort study used patient records from the MIMIC-IV database to examine outcomes in sepsis patients. The primary outcomes were hospital and ICU mortality at 30, 60, and 90 days. The correlation between GPR and these outcomes was evaluated using Kaplan-Meier survival analysis, Cox regression models, and restricted cubic spline (RCS) regression analysis. Sensitivity analyses, including Propensity Score Matching (PSM) and E-value Quantification and Subgroup analyses, were performed to assess the robustness of the findings.

**Results:**

The study included 9,108 patients with sepsis. Kaplan-Meier survival curves indicated progressively worsening survival probabilities from Q1 to Q4 for both hospital and ICU mortality across all time points. Cox analysis revealed that patients in the highest GPR quartile (Q4) had a significantly increased risk of mortality compared to those in the lowest quartile (Q1). A nonlinear relationship between GPR and mortality was identified, with a critical threshold at GPR=30. Subgroup analysis showed that the effect size and direction were consistent across different subgroups. Sensitivity analyses, including E-value quantification and propensity score matching, supported the robustness of our findings.

**Conclusion:**

This study demonstrates that higher GPR levels strongly predict increased short- and long-term mortality risk in ICU-admitted sepsis patients. The composite nature of GPR, reflecting both hyperglycemia and hypokalemia, offers incremental prognostic value beyond single metabolic parameter. A critical threshold effect was observed at GPR=30, where risk substantially increased. This consistent association across patient subgroups positions GPR as a promising biomarker for identifying high-risk sepsis patients, warranting prospective validation.

## 1 Background

Sepsis, a life-threatening organ dysfunction stemming from a dysregulated host response to infection, poses a significant challenge in intensive care units (ICUs) across the globe. Despite advancements in medical care, it remains one of the leading causes of morbidity and mortality, impacting millions annually and resulting in substantial healthcare expenditures (1). The pathophysiology of sepsis is intricate, characterized by a cascade of inflammatory responses that lead to widespread cellular and metabolic abnormalities. Notably, alterations in glucose and potassium homeostasis are critical metabolic disruptions that affect cellular function and systemic homeostasis. Hyperglycemia is frequently observed in septic patients, often attributed to stress-induced hypermetabolism and insulin resistance (2). This metabolic state intensifies oxidative stress and inflammation, further compromising immune function and organ performance. Conversely, potassium imbalances, such as hypokalemia, are common due to factors like increased renal excretion and intracellular shifts caused by insulin therapy or catecholamine surges (3). These electrolyte disturbances can lead to severe complications, including cardiac arrhythmias and muscle weakness (4), thereby worsening the clinical trajectory of sepsis. In recent years, there has been a pressing need to identify reliable prognostic markers to enhance the prediction of sepsis outcomes. While markers like procalcitonin, C-reactive protein, and lactate have shown promise (5), they primarily reflect inflammatory or perfusion-related aspects. Consequently, the identification of novel prognostic biomarkers that capture the complex metabolic imbalances in sepsis remains a crucial research priority.

The serum glucose-potassium ratio (GPR) has emerged as a promising biomarker that reflects the dynamic interplay between glucose and potassium homeostasis, which is often disrupted in various pathological states. Its clinical utility has been recognized in conditions such as diabetic ketoacidosis (6), myocardial infarction (7), and heart failure (8), where it offers a composite view of metabolic derangements that singular parameters fail to capture. In these conditions, an altered GPR has been associated with increased morbidity and mortality, suggesting its potential as a prognostic tool. For instance, studies in myocardial infarction patients have demonstrated a correlation between a high GPR and adverse cardiovascular events, indicating that this biomarker could enhance risk stratification and guide treatment decisions (9, 10). However, research on the application of GPR in sepsis remains limited and has yielded mixed results. Some studies suggest that a high GPR correlates with increased mortality rates and worsened clinical outcomes in sepsis patients, positing that the ratio could serve as an early warning of metabolic decompensation (11, 12). In contrast, a study by Güler et al. (13) found no significant predictive relationship between the glucose-to-potassium ratio and mortality risk in sepsis or septic shock patients admitted to the emergency intensive care unit. These discrepancies may stem from variations in study design, patient populations, or analytical methods. Furthermore, the lack of standardized thresholds and guidelines for interpreting GPR in sepsis complicates its clinical application. Thus, the current understanding of GPR’s relevance to sepsis is limited, underscoring the need for comprehensive evaluations and validation in larger, well-characterized cohorts to establish its potential as a reliable prognostic indicator.

In this context, the MIMIC-IV database serves as a rich repository of de-identified health-related data from thousands of ICU admissions (14), offering a unique opportunity to comprehensively investigate the clinical parameters of sepsis. The database is publicly accessible via the MIMIC-IV platform and contains extensive datasets, including vital signs, laboratory results, and clinical outcomes, which facilitate large-scale retrospective analyses (15). This study aims to explore the association between the serum glucose-potassium ratio and short- and long-term all-cause mortality in ICU-admitted sepsis patients using the MIMIC-IV database. By examining this relationship, we aim to enhance the understanding of metabolic markers in sepsis and potentially identify a novel prognostic indicator that can improve risk stratification and inform treatment strategies for critically ill patients.

## 2 Methods

### 2.1 Data source and study design

We conducted a retrospective cohort study utilizing data from the MIMIC-IV database (version 2.2), which is developed and maintained by the Massachusetts Institute of Technology (MIT) and Beth Israel Deaconess Medical Center (BIDMC) (15). This database comprises two in-house systems: a customized hospital-wide electronic health record (EHR) and an ICU-specific clinical information system, encompassing data from 2008 to 2024. One of the authors (JQ L) completed the necessary authentication process and passed the Collaborative Institutional Training Initiative examination (authentication number 60691748) to access the database. Relevant variables were extracted, and patient data were de-identified to ensure privacy. Given the study’s retrospective nature and the anonymized patient data, the Human Research Ethics Committee of Ningbo No.2 Hospital waived the requirement for informed consent.

### 2.2 Participants

**Figure 1:**
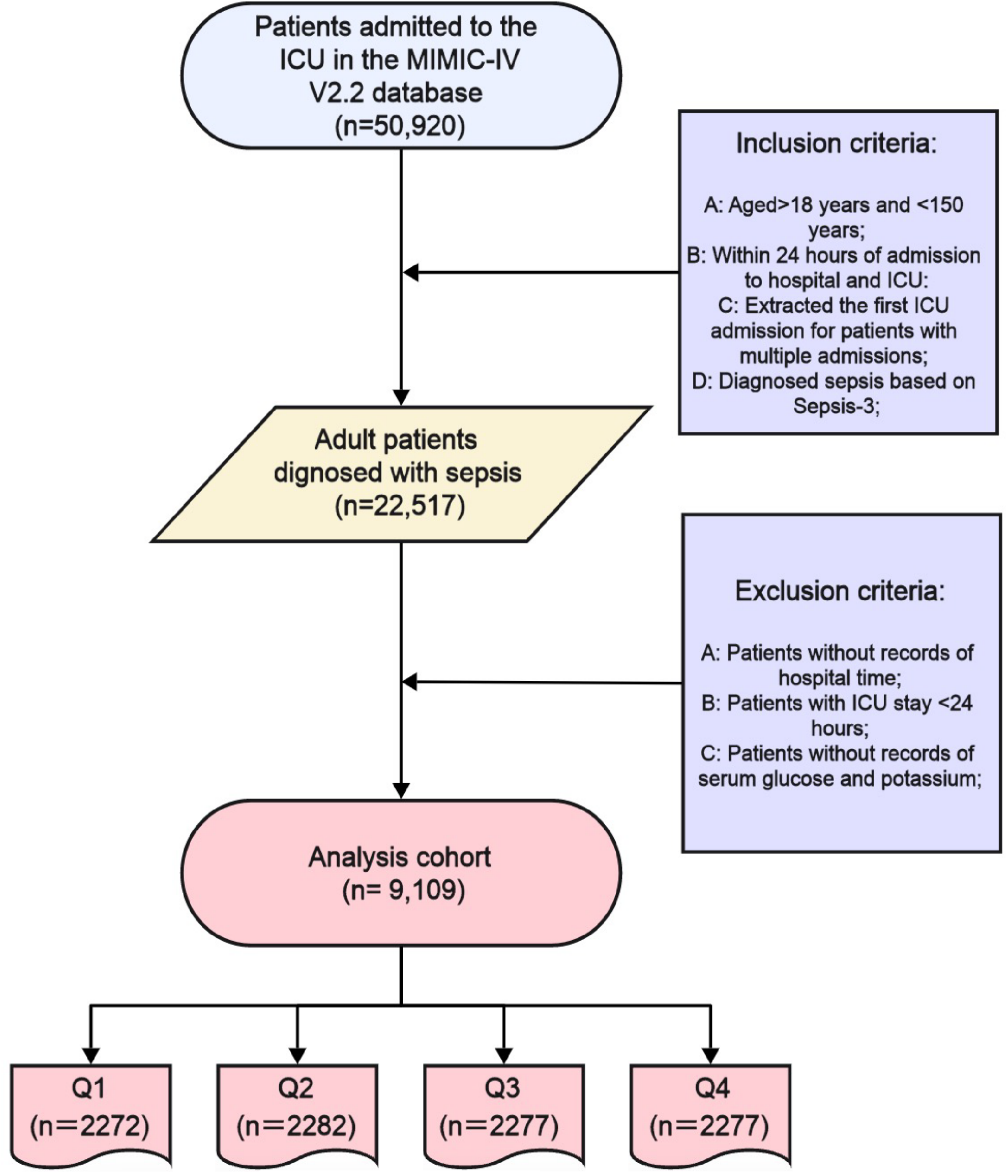
Selection of the study population from the MIMIC-IV database.

The study encompassed all sepsis patients from the MIMIC-IV v2.2 database. Sepsis was defined according to the Sepsis 3.0 criteria, which were jointly established by the American Society for Critical Care Medicine (SCCM) and the European Society for Critical Care Medicine (ESICM). Patient data were extracted using PostgreSQL. The inclusion criteria were sepsis patients aged 18 and above who were admitted to the ICU for the first time. The following exclusion criteria were applied: (1) patients under 18 years old; (2) ICU stay shorter than 48 hours; (3) patients with recurrent sepsis (only their initial ICU admission was considered); and (4) insufficient data, such as missing records for serum glucose and potassium (**Figure 1**).

### 2.3 Research procedures and definitions

Data extraction from MIMIC-IV was performed using Structured Query Language (SQL) via Navicat Premium. The extracted data encompassed a comprehensive set of variables, including patient demographics (age, height, weight, gender, insurance, race, marital status), medical history (hypertension, type 2 diabetes, heart failure, myocardial infarction, malignant tumors, chronic renal failure, cirrhosis, hepatitis, tuberculosis, pneumonia, chronic obstructive pulmonary disease, hyperlipidemia, etc.), and initial laboratory test results (white blood cell count, red blood cell count, neutrophil count, lymphocyte count, platelet count, hemoglobin, mean corpuscular volume, hematocrit, albumin, globulin, total protein, sodium, potassium, calcium, chloride, blood glucose, GPR, anion gap, blood pH, lactate, free calcium, thrombin time, fibrinogen, partial thromboplastin time, international normalized ratio, bilirubin, ALT, AST, urea nitrogen, creatinine, troponin, urine protein, creatine kinase, creatine kinase isoenzyme, N-terminal B-type natriuretic peptide precursor). Special treatments (mechanical ventilation and CRRT), clinical scores (SOFA score, APACHE III score, SAPS II, Oasis score, Charlson Comorbidity Index, SIRS score, GCS score), and clinical outcomes (length of hospital stay, in-hospital mortality, ICU stay, ICU mortality) were also recorded. The 30-day, 60-day, and 90-day mortality rates were calculated. During data cleaning, predictors with more than 30% missing data were excluded. The serum glucose-potassium ratio (GPR) was calculated using the first recorded serum glucose and potassium measurements obtained within 24 hours of ICU admission, based on the formula: GPR = serum glucose (mg/dL)/serum potassium (mmol/L) (16).

### 2.4 Outcomes and measures

The primary outcomes of this study were hospital mortality and ICU mortality at 30-day, 60-day and 90-day.

### 2.5 Statistical analysis

Continuous variables were presented as mean ± standard deviation or median (interquartile range), while categorical variables were reported as frequency and percentage. Data conforming to a normal distribution were analyzed through the t-test or analysis of variance (ANOVA).

For data not following a normal distribution, the Mann-Whitney U test or Kruskal-Wallis test was employed (17, 18). Kaplan-Meier survival analysis was utilized to assess the incidence of endpoint events across different GPR levels, with differences evaluated through the log-rank test. Kaplan-Meier curves offer a visual comparison of survival differences between groups or conditions and do not require prior assumptions about data distribution (19), so it was relatively flexible in use.

The Cox proportional hazards model was utilized to calculate the hazard ratio (HR) and 95% confidence interval (CI) between the GPR and the endpoint. This model, taking survival outcome and survival time as dependent variables, enabled simultaneous analysis of multiple factors affecting survival and analysis of the data with censored survival time, and did not necessitate the estimation of the survival distribution type (20). The GPR was analyzed both as a continuous variable and by quartiles. Cox proportional hazards models were constructed in three sequential tiers: Model 1 (univariate); Model 2 (adjusted for demographics: age, sex, height, weight, insurance, marital status, race); Model 3 (further adjusted for laboratory/clinical covariates: WBC, RBC, RDW, albumin, chloride, ALT, AST, comorbidities [hypertension, diabetes, heart failure, etc.], treatments [CRRT], and severity scores [SOFA, SAPS II, etc.]).

Restricted cubic splines (RCS) used 4 knots placed at the 5th, 35th, 65th, and 95th percentiles. Nonlinearity was tested via the significance of the second spline term. The GPR was incorporated as either a continuous or ordered variable into the model, with the first quartile of the GPR serving as the reference group. The quartile level was used for the calculation of the P-value of the trend. RCS was a non-parametric flexible fitting method that models survival curves by transforming survival times into piecewise functions at individual nodes (21) and can accommodate various types of survival time distributions without excessive assumptions.

Subgroup analyses (22) were conducted to explore potential differences across various subgroups based on age (≤ 70 years and > 70 years), sex, BMI (<27.4 kg/m², 27.4-31.2 kg/m², ≥31.2 kg/m²), age, sex, BMI, hypertension, type 2 diabetes, heart failure, CKD, stroke, AKI, CRRT, and mechanical ventilation, to evaluate the consistency of the GPR’s prognostic value for the primary outcomes. Cox models were also adopted in subgroup analyses to adjust for all variables in the patient’s baseline information.

Sensitivity analyses included: (1) E-values to quantify unmeasured confounding. To evaluate the potential impact of unmeasured confounding on the association between GPR and mortality outcomes, we also calculated E-values using the formula: E-value = RR + √(RR*(RR-1)), where RR is the hazard ratio (HR) derived from Cox regression models. This approach helped assess the robustness of our findings against unmeasured confounding.; (2) Propensity score matching (PSM) (22)). To further assess the robustness of our findings and address potential confounding factors, we conducted a propensity score matching (PSM) analysis. This method helps to reduce selection bias by balancing the distribution of observed covariates between the exposure groups (high GPR group and low GPR group). We defined the high GPR group as patients with GPR above the mean value and the low GPR group as patients with GPR below the mean value. The nearest-neighbor matching method was used to match each patient in the high GPR group with two patients in the low GPR group (1:2 matching), with a caliper width of 0.2 standard errors. Categorical variables were converted into dummy variables for the analysis. For example, marital status was categorized as divorced (1) versus others (0), married (1) versus others (0), and so on. The matching process aimed to create a more balanced comparison group by controlling for key variables such as age, sex, and SOFA score, which are known to influence outcomes in sepsis patients. In the PSM analysis, the balance assessment focuses on comparing the distribution of covariates between the treatment (high GPR) and control (low GPR) groups. The goal of balance assessment is to ensure that these groups are comparable in terms of key covariates, which is crucial for reducing selection bias and enhancing the validity of the study. It is important to note that different outcome variables do not influence the results of balance assessment, as the assessment is solely concerned with the distribution of covariates. Thus, our selection of covariates for balance assessment is based on their potential confounding effects on the relationship between GPR and hospital mortality. This approach ensures that the matched groups are balanced in terms of key covariates, providing a solid foundation for the subsequent analysis of the association between GPR and hospital mortality. After matching, we repeated the Cox regression analysis to assess the association between GPR and hospital mortality. The primary outcome was the all-cause mortality at 30-day, 60-day, and 90-day. The balance of covariates before and after matching was assessed using standardized bias and t-tests. A standardized bias of less than 10% and a p-value greater than 0.05 for the t-tests indicated successful matching. Additionally, a common support test was performed to ensure that the propensity scores of the treatment and control groups overlapped sufficiently, minimizing potential biases.

Data processing and analysis were carried out via R version 4.3.0, along with Zstats v1.0 (www.zstats.net), with statistical significance set at P<0.05 for two-tailed tests. The primary analyses utilized the following packages: Data management and transformation were conducted using dplyr and tidyr. Survival analyses including Kaplan-Meier curves, log-rank tests (via survdiff()), and univariate/multivariate Cox proportional hazards regression (via coxph()) were implemented with the survival package. Nonlinear relationships were assessed through RCS using the rms package, with knots placement and trend significance testing performed via rcs() and anova() functions. Subgroup analyses were streamlined using purrr for iterative modeling and broom for result standardization. E-value analysis was also conducted in R, utilizing packages survival for Cox regression and EValue for E-value calculation. The PSM was performed using the MatchIt package in R, which allows for various matching algorithms, including nearest neighbor, optimal, and full matching. Visualizations were generated with ggplot2 and enhanced using survminer for survival plots. For missing values in the data, the multiple imputation method of the random forest was used to interpolate the missing value data (through the R package “mice”). Features with missing values exceeding 50% were removed before interpolation.

## 3 Results

Among the adult patients in the MIMIC-IV database, a total of 22,517 subjects met the eligibility criteria. From the database, 148 prognostic factors were initially extracted. Following data cleaning, 80 predictors with over 30% missing data were excluded. In the end, 68 forecast factors were included in the model.

### 3.1 Characteristics of included patients

A total of 9,108 people were included in the study, of which 2,272 (24.95%) were in GPR quantile 1 (Q1) group (GPR ≤ 6.67), 2,282 (25.05%) people were in quantile 2 group (6.67 < GPR≤ 25.71), 2,277 people were in quantile 3 group (25.71 < GPR ≤ 40.81), and 2,277 people in quantile 4 group, accounting for 25.00% (GPR > 40.81). IQR is 15.09. The average GPR of all patients was 35.55 ± 16.49. Upon stratification into these four categories, the distribution of each variable across the groups was analyzed. All baseline data are presented in **Table 1** and **Supplementary Table 1**.

**Table 1:** Summary of characteristics that are statistically different of the study population.

Variables	Total (n = 9,108)	Q1 (n = 2,272)	Q2 (n = 2,282)	Q3 (n = 2,277)	Q4 (n = 2,277)	Statistic	*P*
*Characteristics*							
Age (year)	71.61 ± 14.73	72.07 ± 14.94	71.52 ± 14.98	71.95 ± 14.52	70.91 ± 14.45	F=2.91	**0.033**
Weight (kg)	79.16 ± 23.62	77.69 ± 23.65	77.85 ± 23.39	78.96 ± 22.97	82.15 ± 24.17	F=17.45	**<0.001**
Gender (n(%))						χ²=13.82	**0.003**
F	4038 (44.33)	944 (41.55)	993 (43.51)	1046 (45.94)	1055 (46.33)		
M	5070 (55.67)	1328 (58.45)	1289 (56.49)	1231 (54.06)	1222 (53.67)		
Marital Status, n(%)						χ²=51.12	**<0.001**
Divorced	644 (7.07)	165 (7.26)	163 (7.14)	168 (7.38)	148 (6.50)		
Married	3801 (41.73)	944 (41.55)	960 (42.07)	937 (41.15)	960 (42.16)		
NA	1017 (11.17)	200 (8.80)	229 (10.04)	264 (11.59)	324 (14.23)		
Single	2127 (23.35)	573 (25.22)	564 (24.72)	497 (21.83)	493 (21.65)		
Widowed	1519 (16.68)	390 (17.17)	366 (16.04)	411 (18.05)	352 (15.46)		
*Laboratory parameters*							
WBC (×10^9^/L)	13.76 ± 12.36	13.37 ± 13.52	13.08 ± 11.54	13.72 ± 9.73	14.88 ± 14.11	F=9.27	**<0.001**
RBC (×10^12^/L)	3.42 ± 0.70	3.32 ± 0.68	3.38 ± 0.66	3.46 ± 0.71	3.51 ± 0.73	F=35.03	**<0.001**
Hemoglobin (g/dL)	10.23 ± 1.97	9.93 ± 1.90	10.15 ± 1.86	10.36 ± 2.01	10.47 ± 2.07	F=34.01	**<0.001**
RDW (%)	16.00 ± 2.51	16.48 ± 2.63	15.96 ± 2.47	15.88 ± 2.50	15.67 ± 2.36	F=43.44	**<0.001**
Hematocrit (%)	31.29 ± 5.92	30.60 ± 5.83	30.95 ± 5.52	31.60 ± 6.01	32.02 ± 6.19	F=26.51	**<0.001**
Albumin (g/L)	2.91 ± 0.65	2.86 ± 0.65	2.89 ± 0.64	2.96 ± 0.67	2.93 ± 0.65	F=4.76	**0.003**
Sodium (mmol/L)	138.56 ± 5.76	137.62 ± 5.44	138.56 ± 5.55	138.81 ± 5.43	139.24 ± 6.44	F=32.59	**<0.001**
Potassium (mmol/L)	4.26 ± 0.64	4.60 ± 0.68	4.25 ± 0.58	4.14 ± 0.57	4.06 ± 0.59	F=344.80	**<0.001**
Chlorine (mmol/L)	104.06 ± 7.03	103.48 ± 6.87	104.42 ± 6.73	104.20 ± 6.74	104.12 ± 7.69	F=7.51	**<0.001**
Glucose (mmol/L)	148.62 ± 64.05	96.47 ± 18.25	122.39 ± 17.48	148.66 ± 23.13	226.90 ± 75.54	F=4205.85	**<0.001**
Anion gap (mmol/L)	15.73 ± 4.61	15.99 ± 4.95	14.97 ± 4.21	15.33 ± 4.21	16.65 ± 4.85	F=60.36	**<0.001**
pH	7.35 ± 0.09	7.34 ± 0.09	7.36 ± 0.08	7.36 ± 0.08	7.35 ± 0.10	F=32.84	**<0.001**
PCO_2_ (mmHg)	41.76 ± 11.39	42.69 ± 12.88	41.85 ± 10.96	41.51 ± 11.13	41.13 ± 10.57	F=5.78	**<0.001**
PO_2_ (mmHg)	118.76 ± 71.09	113.39 ± 72.25	122.64 ± 73.70	121.03 ± 69.82	117.69 ± 68.65	F=5.45	**<0.001**
Free calcium (mmol/L)	1.10 ± 0.11	1.11 ± 0.11	1.11 ± 0.10	1.10 ± 0.10	1.10 ± 0.11	F=3.67	**0.012**
PT (s)	18.31 ± 10.24	19.64 ± 11.10	17.67 ± 8.72	17.64 ± 9.14	18.31 ± 11.56	F=17.03	**<0.001**
Fibrinogen (mg/dL)	310.32 ± 187.88	287.49 ± 173.05	291.86 ± 171.25	335.99 ± 201.95	330.48 ± 201.03	F=11.33	**<0.001**
PPT (s)	40.89 ± 20.28	40.87 ± 17.61	39.63 ± 18.68	40.39 ± 20.54	42.65 ± 23.60	F=8.20	**<0.001**
INR	1.68 ± 0.99	1.82 ± 1.12	1.62 ± 0.86	1.63 ± 0.97	1.66 ± 1.00	F=18.18	**<0.001**
Total bilirubin (mg/dL)	2.79 ± 5.88	3.67 ± 6.79	2.78 ± 5.87	2.84 ± 6.08	1.92 ± 4.47	F=22.47	**<0.001**
Direct bilirubin (mg/dL)	4.24 ± 5.73	5.05 ± 6.18	4.33 ± 5.84	4.09 ± 5.52	3.29 ± 5.12	F=2.88	**0.035**
Indirect bilirubin (mg/dL)	2.22 ± 2.85	2.65 ± 3.45	2.45 ± 2.83	1.85 ± 2.32	1.86 ± 2.46	F=3.36	**0.019**
ALT (U/L)	167.94 ± 596.01	186.59 ± 784.00	134.50 ± 479.30	140.58 ± 452.44	205.54 ± 597.57	F=5.00	**0.002**
AST (U/L)	302.91 ± 1065.00	349.66 ± 1235.43	253.35 ± 858.19	234.05 ± 758.68	366.35 ± 1273.72	F=5.79	**<0.001**
Urea nitrogen (mmol/L)	35.33 ± 25.42	39.73 ± 27.33	32.31 ± 24.14	32.67 ± 23.03	36.64 ± 26.23	F=44.41	**<0.001**
Creatinine (mg/dL)	1.77 ± 1.60	2.17 ± 1.96	1.57 ± 1.39	1.61 ± 1.47	1.74 ± 1.42	F=70.06	**<0.001**
LDH (U/L)	715.76 ± 1616.90	828.65 ± 2105.51	633.76 ± 1211.17	563.17 ± 1040.05	829.50 ± 1828.93	F=7.34	**<0.001**
CKMB (U/L)	21.15 ± 52.72	14.24 ± 34.27	17.83 ± 48.56	21.76 ± 51.27	28.24 ± 65.76	F=12.94	**<0.001**
Troponint (μg/L)	0.75 ± 2.38	0.39 ± 1.15	0.61 ± 1.71	0.71 ± 2.06	1.14 ± 3.43	F=16.66	**<0.001**
NT-proBNP (pmol/L)	10065.50 ± 12234.37	11888.63 ± 12317.15	8313.65 ± 10569.28	9541.94 ± 12367.29	10538.55 ± 13343.72	F=2.69	**0.045**
*Treatment*							
CRRT (n(%))						χ²=18.04	**<0.001**
No	8297 (91.10)	2026 (89.17)	2112 (92.55)	2091 (91.83)	2068 (90.82)		
Yes	811 (8.90)	246 (10.83)	170 (7.45)	186 (8.17)	209 (9.18)		
Ventilation (hours)	101.88 ± 145.10	91.36 ± 144.60	99.46 ± 141.63	108.83 ± 152.51	107.59 ± 140.66	F=5.94	**<0.001**
*Comorbidity*							
Hypertension (n(%))						χ²=41.33	**<0.001**
No	5615 (61.65)	1526 (67.17)	1394 (61.09)	1350 (59.29)	1345 (59.07)		
Yes	3493 (38.35)	746 (32.83)	888 (38.91)	927 (40.71)	932 (40.93)		
Type 2 diabetes mellitus (n(%))						χ²=640.05	**<0.001**
No	6235 (68.46)	1760 (77.46)	1787 (78.31)	1599 (70.22)	1089 (47.83)		
Yes	2873 (31.54)	512 (22.54)	495 (21.69)	678 (29.78)	1188 (52.17)		
Myocardial infarct (n(%))						χ²=50.89	**<0.001**
No	8397 (92.19)	2139 (94.15)	2130 (93.34)	2104 (92.40)	2024 (88.89)		
Yes	711 (7.81)	133 (5.85)	152 (6.66)	173 (7.60)	253 (11.11)		
Malignant tumor (n(%))						χ²=35.32	**<0.001**
No	7061 (77.53)	1719 (75.66)	1720 (75.37)	1758 (77.21)	1864 (81.86)		
Yes	2047 (22.47)	553 (24.34)	562 (24.63)	519 (22.79)	413 (18.14)		
Chronic kidney diseases (n(%))						χ²=17.68	**<0.001**
No	6884 (75.58)	1663 (73.20)	1776 (77.83)	1753 (76.99)	1692 (74.31)		
Yes	2224 (24.42)	609 (26.80)	506 (22.17)	524 (23.01)	585 (25.69)		
Acute renal failure (n(%))						χ²=27.54	**<0.001**
No	4374 (48.02)	1011 (44.50)	1188 (52.06)	1106 (48.57)	1069 (46.95)		
Yes	4734 (51.98)	1261 (55.50)	1094 (47.94)	1171 (51.43)	1208 (53.05)		
Cirrhosis (n(%))						χ²=42.28	**<0.001**
No	7998 (87.81)	1934 (85.12)	1979 (86.72)	2009 (88.23)	2076 (91.17)		
Yes	1110 (12.19)	338 (14.88)	303 (13.28)	268 (11.77)	201 (8.83)		
Stroke (n(%))						χ²=25.50	**<0.001**
No	8138 (89.35)	2068 (91.02)	2054 (90.01)	2043 (89.72)	1973 (86.65)		
Yes	970 (10.65)	204 (8.98)	228 (9.99)	234 (10.28)	304 (13.35)		
Hyperlipidemia, (n(%))						χ²=37.19	**<0.001**
No	6215 (68.24)	1628 (71.65)	1572 (68.89)	1570 (68.95)	1445 (63.46)		
Yes	2893 (31.76)	644 (28.35)	710 (31.11)	707 (31.05)	832 (36.54)		
Acute kidney injury stage (n(%))						χ²=34.38	**<0.001**
1	1403 (18.94)	356 (19.63)	347 (18.89)	353 (18.76)	347 (18.51)		
2	3087 (41.67)	666 (36.71)	835 (45.45)	810 (43.04)	776 (41.39)		
3	2918 (39.39)	792 (43.66)	655 (35.66)	719 (38.20)	752 (40.11)		
*Scoring systems*							
SOFA score (score)	6.77 ± 3.90	7.15 ± 4.08	6.23 ± 3.65	6.52 ± 3.74	7.18 ± 4.02	F=33.60	**<0.001**
APSIII score (score)	58.48 ± 23.59	60.89 ± 24.31	53.75 ± 21.65	56.23 ± 21.93	63.08 ± 25.13	F=76.14	**<0.001**
SAPSII score (score)	45.83 ± 14.75	47.50 ± 15.57	43.74 ± 13.71	44.94 ± 14.03	47.14 ± 15.28	F=34.07	**<0.001**
OASIS, score (score)	36.09 ± 8.90	36.05 ± 8.95	35.07 ± 8.57	35.91 ± 8.70	37.32 ± 9.24	F=24.98	**<0.001**
GCS score (score)	13.06 ± 3.18	13.12 ± 3.08	13.23 ± 2.90	13.07 ± 3.15	12.84 ± 3.53	F=6.07	**<0.001**
Charlson score (score)	6.53 ± 2.81	6.69 ± 2.81	6.42 ± 2.76	6.42 ± 2.79	6.59 ± 2.87	F=5.23	**<0.001**
SIRS score (score)						χ²=110.42	**<0.001**
0	60 (0.66)	18 (0.79)	19 (0.83)	13 (0.57)	10 (0.44)		
1	637 (6.99)	188 (8.27)	187 (8.19)	145 (6.37)	117 (5.14)		
2	2302 (25.27)	655 (28.83)	629 (27.56)	549 (24.11)	469 (20.60)		
3	3837 (42.13)	916 (40.32)	939 (41.15)	993 (43.61)	989 (43.43)		
4	2272 (24.95)	495 (21.79)	508 (22.26)	577 (25.34)	692 (30.39)		

Continuous variables are expressed as the median and interquartile range. Counting data are presented as numbers and percentages. The medical condition was defined based on the ICD-9 code. WBC, white blood cell; RBC, red blood cell; RDW, red blood cell distribution width; PCO_2_, partial pressure of carbon dioxide; PO_2_, partial pressure of oxygen; LD, Lactate Dehydrogenase; PT, prothrombin time; PTT, partial thromboplastin time; INR, international normalized ratio; ALT, alanine aminotransferase; AST, aspartate aminotransferas; CKMB, creatine kinase-MB; BCK, blood ketone; NT-proBNP, N-terminal pro-brain natriuretic peptide; CRRT, continuous renal replacement therapy; COPD, chronic obstructive pulmonary disease; OASIS, oxford acute severity of illness score; SASPII, simplified acute physiology score II; SOFA, sequential organ failure assessment; CNS, central nervous system; GCS, Glasgow Coma Scale; SIRS, Systemic Inflammatory Response Syndrome; F, ANOVA; χ², Chi-square test; SD, standard deviation. Bold red font indicates p-values with statistical significance.

Patients in Q1 were older and had lower body weight than those in the other groups, and there were differences in sex and marital status among the four groups. WBC, RBC, platelet, hemoglobin, hematocrit, albumin, sodium, glucose, anion gap, fibrinogen, PPT, ALT, CK, CKMB were also higher in Q4 group than in Q1 group, but RDW, potassium, hematocrit and bilirubin were lower than Q1 group. There was no significant difference in height, insurance, languages, CRRT days, ventilation, COPD, heart failure, hepatitis, tuberculosis and pneumonia (P>0.05) (**Table 1**).

### 3.2 Kaplan-Meier survival curve analysis

**Figure 2:**
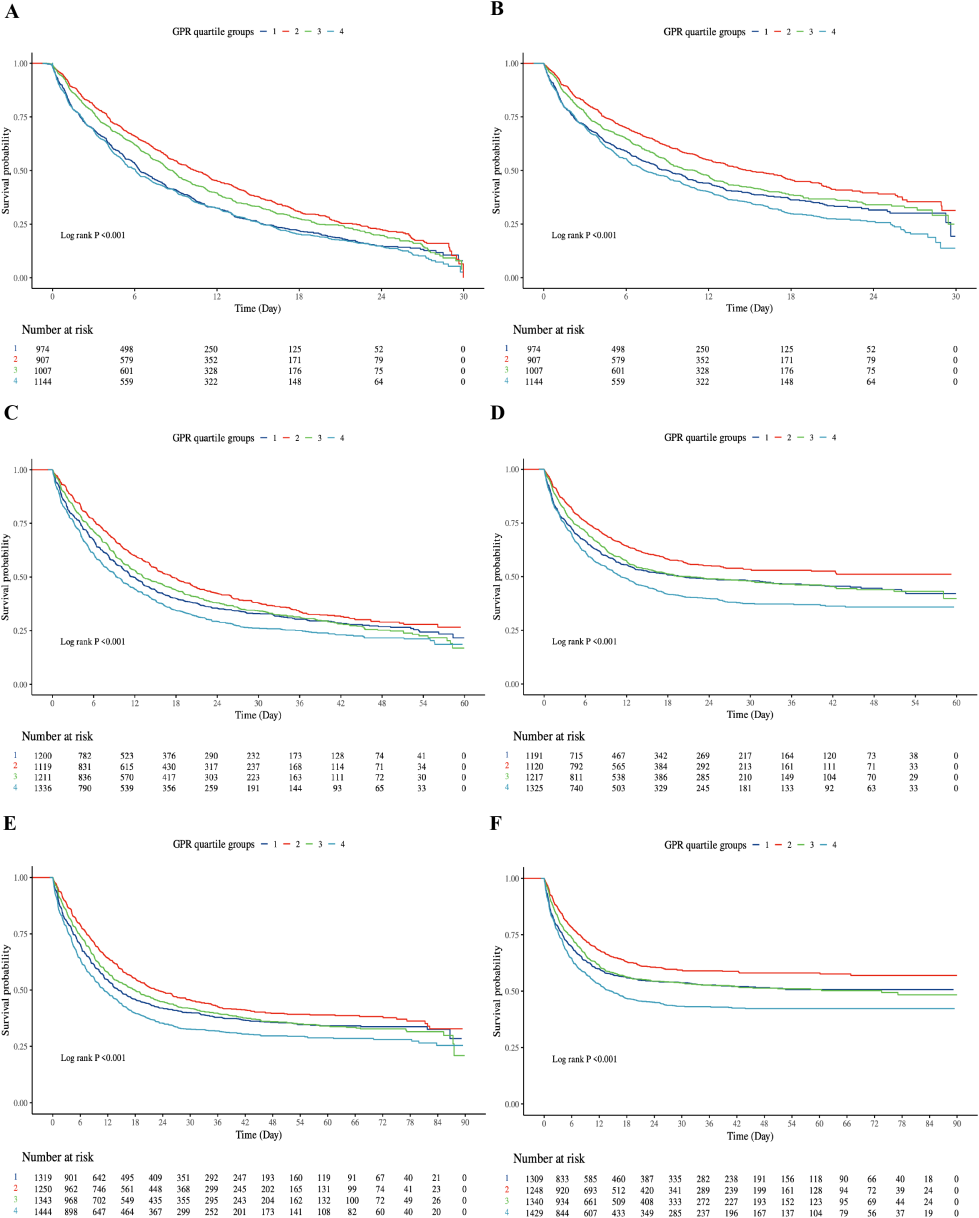
Kaplan-Meier survival curve of cumulative survival rate during hospitalization and ICU for groups. **(A)**: Kaplan-Meier survival curve of cumulative survival rate during hospitalization for groups at 30-day. **(B)**: Kaplan-Meier survival curve of cumulative survival rate during ICU for groups at 30-day. **(C)** Kaplan-Meier survival curve of cumulative survival rate during hospitalization for groups at 60-day. **(D)** Kaplan-Meier survival curve of cumulative survival rate during ICU for groups at 60-day. **(E)** Kaplan-Meier survival curve of cumulative survival rate during hospitalization for groups at 90-day. **(F)** Kaplan-Meier survival curve of cumulative survival rate during ICU for groups at 90-day. X-axis: Time (Days); Y-axis: Survival Probability. Log-rank test, all P < 0.001. Q1: dark blue; Q2: red; Q3: green; Q4: light blue.

Kaplan-Meier curves (**Figure 2**) demonstrated worsening survival probabilities from Q1 to Q4 for both hospital and ICU mortality at 30-day, 60-day, and 90-day intervals (log-rank test, all P < 0.001). Specifically, a total of 9,108 people were included in the study, of which 2,272 (24.95%) were in GPR quantile 1 (Q1) group (GPR ≤ 6.67), 2,282 (25.05%) people were in quantile 2 group (6.67 < GPR≤ 25.71), 2,277 people were in quantile 3 group (25.71 < GPR ≤ 40.81), and 2,277 people in quantile 4 group, accounting for 25.00% (GPR > 40.81).

### 3.3 Cox regression models for all-cause mortality (in hospital and ICU)

In the Cox regression analysis, a higher GPR was positively correlated with increased mortality rates in both the ICU and hospital settings among critically ill patients with sepsis. When the GPR was analyzed as a continuous variable, it was independently associated with a higher risk of hospital mortality both at 30-day, 60-day and 90-day (All P < 0.05). Patients in Q4 had a 15–20% higher risk of mortality compared to Q1 across all time points. At 60-day, when categorized into quartiles, Model 1 revealed that the risk of hospital mortality for Q4 were 19% higher than for Q1 (HR 1.19 [95% CI 1.08 to 1.31], P < 0.001), Model 2 revealed that the risk of hospital mortality for Q4 were 18% higher than for Q1 (HR 1.18 [95% CI 1.03 to 1.35], P < 0.001). At 90-day, when categorized into quartiles, Model 1 revealed that the risk of hospital mortality for Q4 were 20% higher than for Q1 (HR 1.20 [95% CI 1.09 to 1.32], P < 0.001), Model 2 revealed that the risk of hospital mortality for Q4 were 15% higher than for Q1 (HR 1.15 [95% CI 1.01 to 1.32], P = 0.037). The differences in Model 3 results compared to Models 1 and 2 are likely due to the additional adjustment for confounding variables such as WBC, RBC, RDW, albumin, chloride, ALT, etc.

For ICU mortality, the GPR, when used as a continuous variable, was significantly associated with an elevated risk of ICU death in Models 1, 2 and 3 (All P < 0.001). Furthermore, when the GPR was categorized into quartiles, at 30-day, Model 1 demonstrated that the risk of ICU mortality for Q4 was 1.13 times that of Q1 (HR 1.13 [95% CI 1.01 to 1.26], P < 0.001). At 60-day, Model 1 demonstrated that the risk of ICU mortality for Q4 was 1.23 times that of Q1 (HR 1.23 [95% CI 1.10 to 1.37], P < 0.001), Model 2 demonstrated that the risk of ICU mortality for Q4 was 1.21 times that of Q1 (HR 1.04 [95% CI 1.01 to 1.41], P = 0.015) (**Table 2**).

**Table 2:** The association between GPR groups and in-hospital and ICU mortality at 30-day, 60-day and 90-day.

Exposure	Model 1		Model 2		Model 3	
	HR (95% CI)	*P*-value	HR (95% CI)	*P*-value	HR (95% CI)	*P*-value
In-hospital mortality						
*At 30-day*						
GPR as continuous	1.01 (1.01 ~ 1.01)	**<0.001**	1.01 (1.01 ~ 1.01)	**0.012**	1.01 (1.01 ~ 1.01)	**0.012**
Q1	1.00 (Reference)		1.00 (Reference)		1.00 (Reference)	
Q2	0.73 (0.65 ~ 0.81)	**<0.001**	0.79 (0.68 ~ 0.91)	**0.002**	0.87 (0.70 ~ 1.08)	**0.200**
Q3	0.82 (0.74 ~ 0.91)	**<0.001**	0.81 (0.70 ~ 0.93)	**0.004**	0.80 (0.64 ~ 0.99)	**0.042**
Q4	1.05 (0.95 ~ 1.15)	0.370	0.99 (0.86 ~ 1.13)	0.838	0.99 (0.80 ~ 1.22)	0.916
*At 60-day*						
GPR as continuous	1.01 (1.01 ~ 1.01)	**<0.001**	1.01 (1.01 ~ 1.01)	**<0.001**	1.01 (1.01 ~ 1.01)	**0.012**
Q1	1.00 (Reference)		1.00 (Reference)		1.00 (Reference)	
Q2	0.81 (0.73 ~ 0.90)	**<0.001**	0.92 (0.80 ~ 1.07)	0.270	0.91 (0.74 ~ 1.13)	0.397
Q3	0.94 (0.85 ~ 1.04)	0.214	0.98 (0.85 ~ 1.13)	0.795	0.92 (0.75 ~ 1.13)	0.433
Q4	1.19 (1.08 ~ 1.31)	**<0.001**	1.18 (1.03 ~ 1.35)	**0.015**	1.14 (0.93 ~ 1.41)	0.202
*At 90-day*						
GPR as continuous	1.01 (1.01 ~ 1.01)	**<0.001**	1.01 (1.01 ~ 1.01)	**<0.001**	1.01 (1.01 ~ 1.01)	**0.012**
Q1	1.00 (Reference)		1.00 (Reference)		1.00 (Reference)	
Q2	0.81 (0.73 ~ 0.90)	**<0.001**	0.90 (0.78 ~ 1.03)	0.135	0.87 (0.70 ~ 1.07)	0.173
Q3	0.93 (0.85 ~ 1.03)	0.182	0.96 (0.83 ~ 1.10)	0.523	0.91 (0.74 ~ 1.12)	0.355
Q4	1.20 (1.09 ~ 1.32)	**<0.001**	1.15 (1.01 ~ 1.32)	**0.037**	1.10 (0.90 ~ 1.35)	0.351
ICU mortality						
*At 30-day*						
GPR as continuous	1.01 (1.01 ~ 1.01)	**<0.001**	1.01 (1.01 ~ 1.01)	**<0.001**	1.01 (1.01 ~ 1.01)	**<0.001**
Q1	1.00 (Reference)		1.00 (Reference)		1.00 (Reference)	
Q2	0.72 (0.63 ~ 0.81)	**<0.001**	0.81 (0.69 ~ 0.96)	**0.017**	0.73 (0.65 ~ 0.81)	**<0.001**
Q3	0.87 (0.78 ~ 0.98)	**0.024**	0.85 (0.72 ~ 1.00)	0.052	0.82 (0.74 ~ 0.91)	**<0.001**
Q4	1.13 (1.01 ~ 1.26)	**0.027**	1.07 (0.91 ~ 1.24)	0.425	1.05 (0.95 ~ 1.15)	0.370
*At 60-day*						
GPR as continuous	1.01 (1.01 ~ 1.01)	**<0.001**	1.01 (1.01 ~ 1.01)	**<0.001**	1.01 (1.01 ~ 1.01)	**0.012**
Q1	1.00 (Reference)		1.00 (Reference)		1.00 (Reference)	
Q2	0.76 (0.67 ~ 0.86)	**<0.001**	0.89 (0.76 ~ 1.06)	0.190	0.73 (0.65 ~ 0.81)	**<0.001**
Q3	0.93 (0.83 ~ 1.05)	0.255	0.96 (0.82 ~ 1.13)	0.618	0.82 (0.74 ~ 0.91)	**<0.001**
Q4	1.23 (1.10 ~ 1.37)	**<0.001**	1.21 (1.04 ~ 1.41)	**0.015**	1.05 (0.95 ~ 1.15)	0.370
*At 90-day*						
GPR as continuous	1.01 (1.01 ~ 1.01)	**<0.001**	1.01 (1.01 ~ 1.01)	**<0.001**	1.01 (1.01 ~ 1.01)	**0.012**
Q1	1.00 (Reference)		1.00 (Reference)		1.00 (Reference)	
Q2	0.76 (0.67 ~ 0.86)	**<0.001**	0.88 (0.75 ~ 1.04)	0.148	0.73 (0.65 ~ 0.81)	**<0.001**
Q3	0.95 (0.84 ~ 1.06)	0.353	0.95 (0.81 ~ 1.11)	0.515	0.82 (0.74 ~ 0.91)	**<0.001**
Q4	1.26 (1.13 ~ 1.40)	**<0.001**	1.20 (1.03 ~ 1.40)	**0.018**	1.05 (0.95 ~ 1.15)	0.370

*GPR: Q1 (Quartile 1; GPR ≤ 6.67, n=436), Q2 (Quartile 2; 6.67 < GPR ≤ 25.71), Q3 (Quartile 3; 25.71 < GPR ≤ 40.81) and Q4 (Quartile 4; GPR > 40.81). HR: hazard ratio; CI: confidential interval.

Model 1: Cox univariate analysis.

Model 2: Adjusted for age, gender, height, weight, insurance, marital status and race.

Model 3: Adjusted for age, gender, height, weight, insurance, marital status and race, WBC, RBC, RDW, albumin, chloride, ALT, AST, Hypertension, Type 2 diabetes mellitus, heart failure, malignant tumor, chronic kidney disease, acute renal failure, stroke, hyperlipidemia, chronic obstructive pulmonary disease, SIRS, CRRT, Oxford acute severity of illness score, Simplified acute physiology score II, Sequential organ failure assessment, Central nervous system, Glasgow coma scale. Bold red font indicates p-values with statistical significance.

### 3.4 RCS regression models for all-cause mortality (in hospital and ICU)

**Figure 3:**
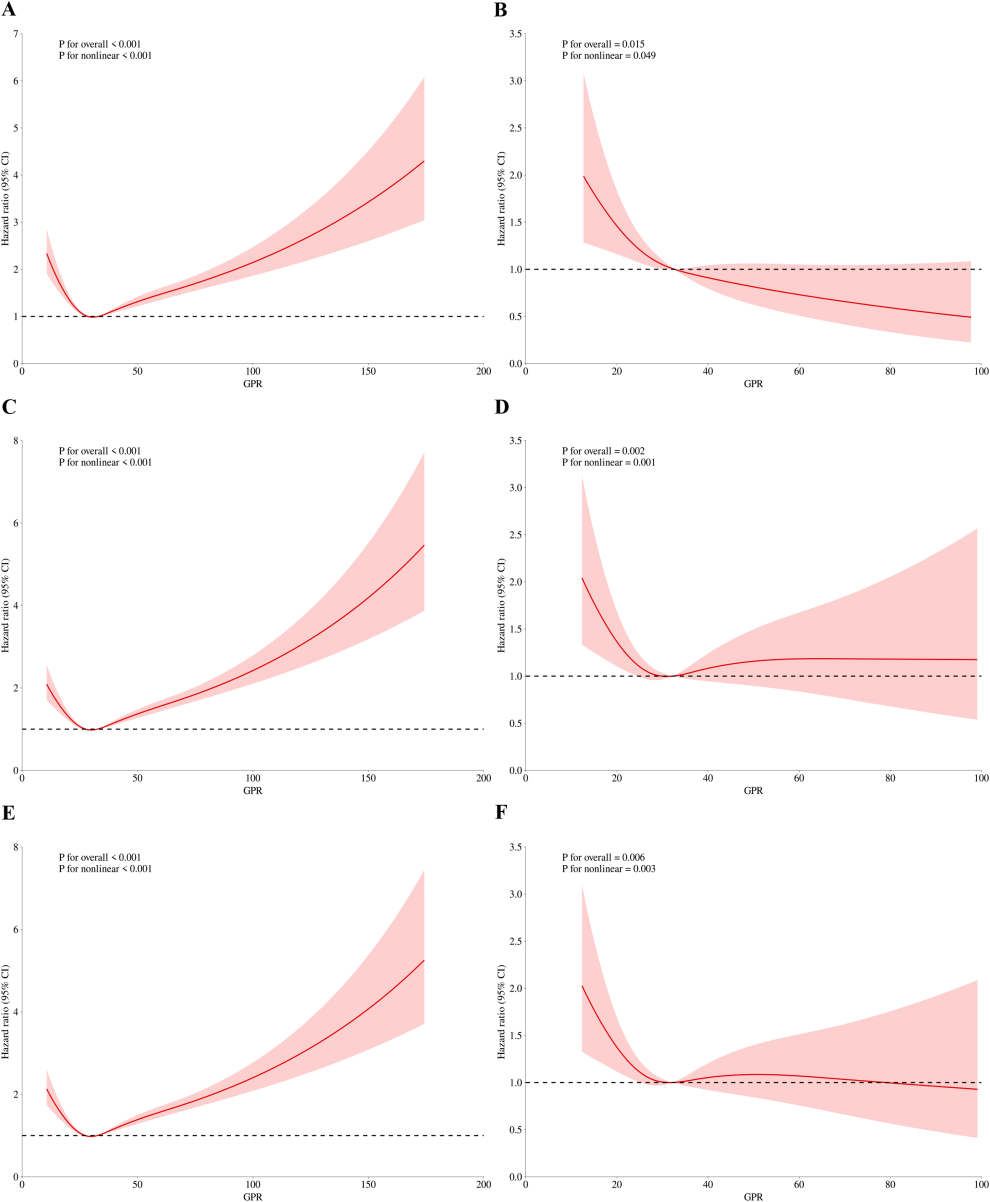
RCS regression for GPR and in-hospital mortality. **(A)** Univariate analysis at 30-day (P for overall effect <0.001; P for nonlinearity <0.001). **(B)** Multivariate analysis at 30-day (P for overall effect <0.001; P for nonlinearity <0.001). **(C)** Univariate analysis at 60-day (P for overall effect <0.001; P for nonlinearity <0.001). **(D)** Multivariate analysis at 60-day (P for overall effect 0.007; P for nonlinearity 0.004). **(E)** Univariate analysis at 90-day (P for overall effect <0.001; P for nonlinearity <0.001). **(F)** Multivariate analysis at 90-day (P for overall effect 0.014; P for nonlinearity 0.010).

**Figure 4:**
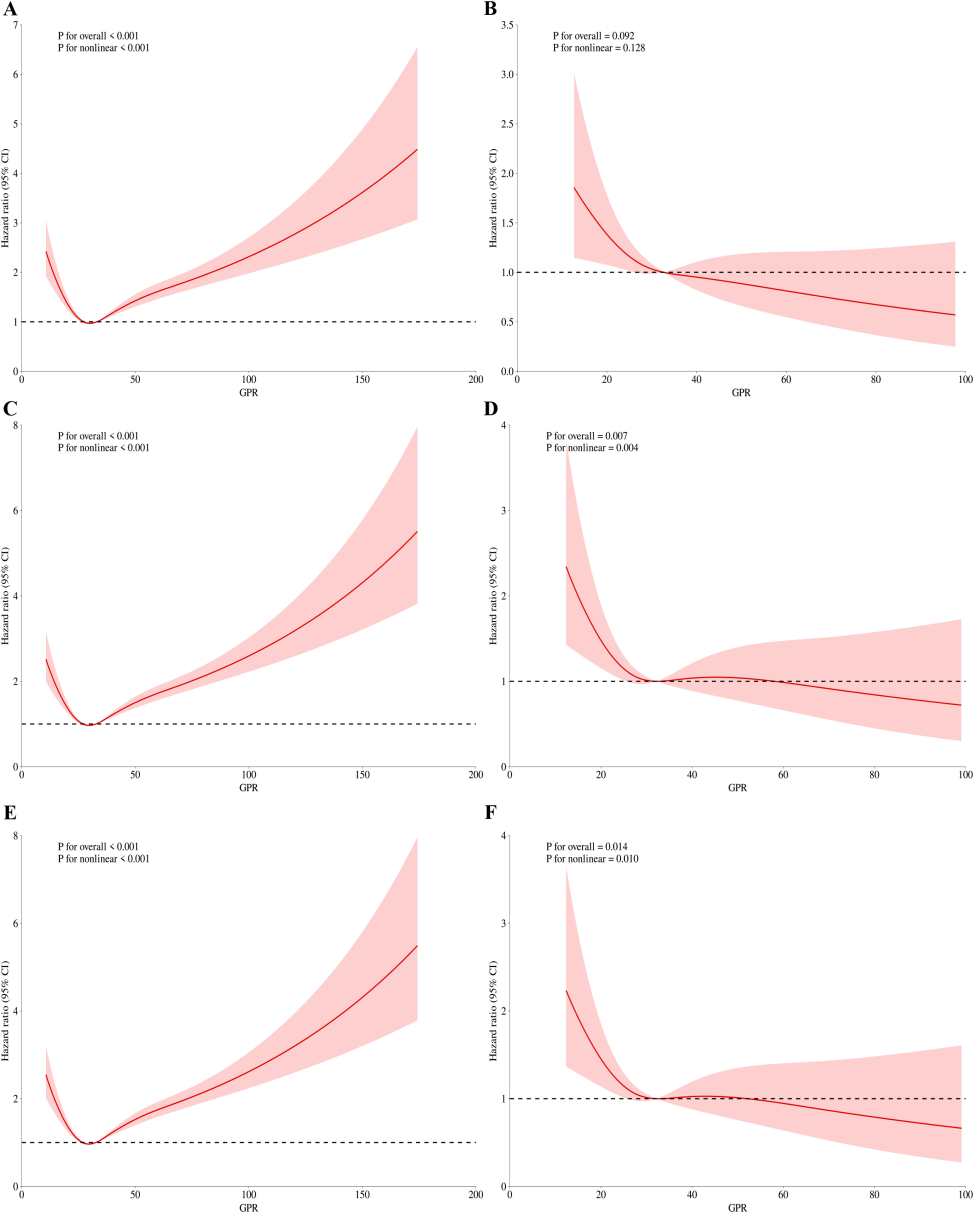
RCS regression for GPR and mortality during ICU admission. **(A)** Univariate analysis at 30-day (P for overall effect <0.001; P for nonlinearity <0.001). **(B)** Multivariate analysis at 30-day (P for overall effect <0.001; P for nonlinearity <0.001). **(C)** Univariate analysis at 60-day (P for overall effect <0.001; P for nonlinearity <0.001). **(D)** Multivariate analysis at 60-day (P for overall effect 0.007; P for nonlinearity 0.004). **(E)** Univariate analysis at 90-day (P for overall effect <0.001; P for nonlinearity <0.001). **(F)** Multivariate analysis at 90-day (P for overall effect 0.014; P for nonlinearity 0.010).

We subsequently employed the RCS regression models to elucidate the risk and discovered a nonlinear association between the GPR and mortality. **Figures 3** and **4** illustrate the results of the univariate and multivariate analyses regarding the relationship between the GPR and in-hospital, In-ICU mortality in three time points, respectively.

**Figures 3A, B** present the findings of the univariate and multivariate analyses concerning the association between the GPR and hospital mortality on 30-day, respectively. Before adjusting for 30-day in-hospital mortality, the p-value for the overall effect was < 0.001, and the p-value for the nonlinear effect was also < 0.001. Following adjustment, all p-values were less than 0.05. Similarly, nonlinear associations were observed for 60-day (**Figures 3C, D**) and 90-day (**Figures 3E, F**) in-hospital mortality, both before and after adjustment for relevant factors.

For ICU mortality, on 30-day mortality (**Figures 4A, B**), the unadjusted p value for the overall effect was less than 0.001, the p value for the nonlinear effect was less than 0.001, and all adjusted p values were greater than 0.05. The unadjusted p value was less than 0.001 for the overall effect and less than 0.001 for the nonlinear effect on 60-day mortality (**Figures 4C, D**). The adjusted p value was 0.007 for the overall effect and 0.004 for the nonlinear effect. Finally, on 90-day mortality (**Figures 4E, F**), the unadjusted p value was less than 0.001 for the overall effect and less than 0.001 for the nonlinear effect. After adjustment, the p value of overall effect was 0.014, and the p value of nonlinear effect was 0.01. **Figures 3** and **4** demonstrate that the inflection point in both multifactorial models is about 30.

### 3.5 Subgroup analysis

In subgroup analyses, the directionality of the effect estimates in subgroups was consistent with the overall outcomes. Subgroup analyses were stratified by age, sex, BMI, hypertension, type 2 diabetes, heart failure, CKD, stroke, AKI, CRRT, and mechanical ventilation.

**Figure 5:**
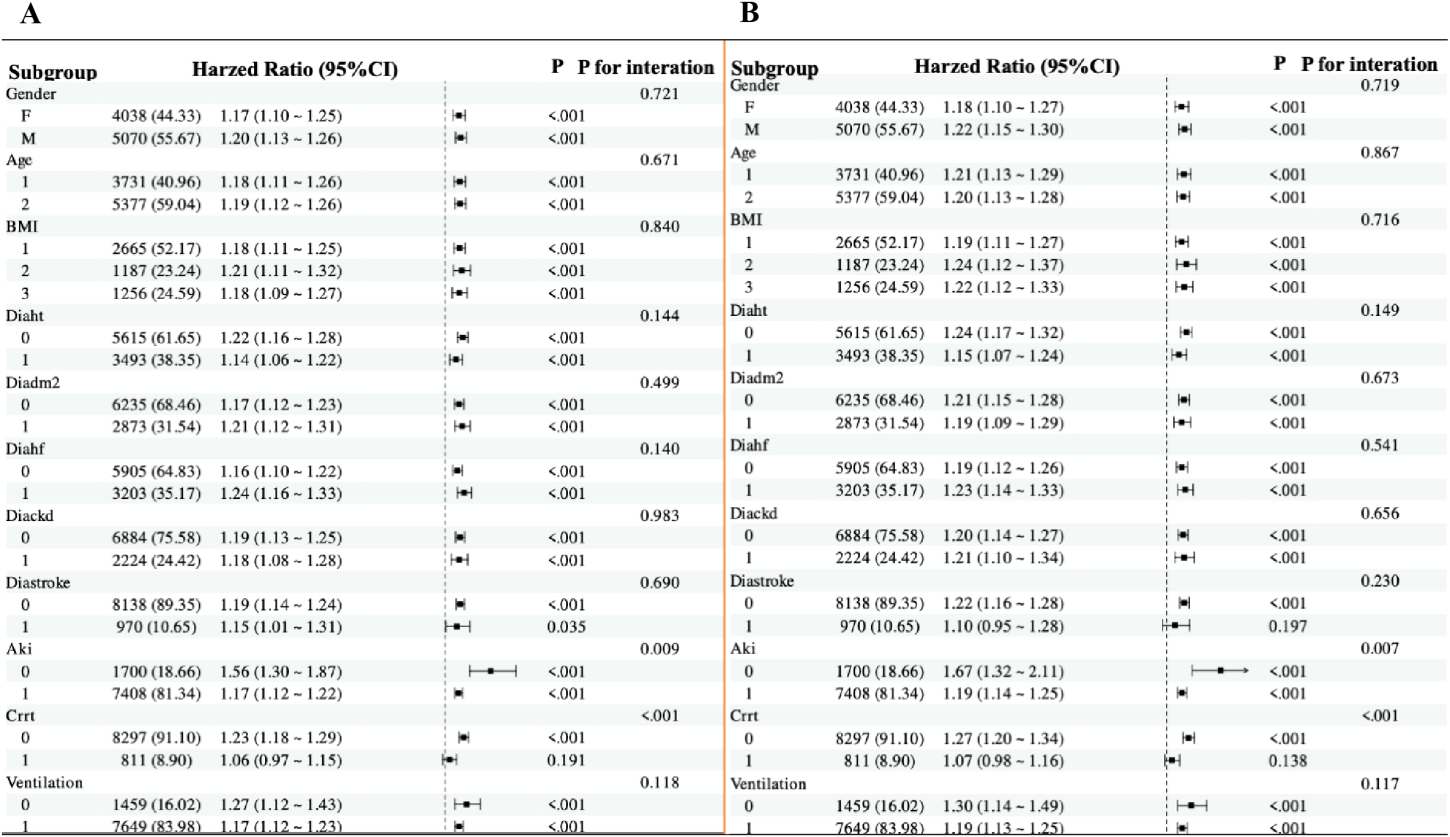
Forest plots for subgroup analyses of the association between GPR and mortality. **(A)** Subgroup analysis of the association between GPR and in-hospital mortality after covariate adjustment. **(B)** Subgroup analysis of the association between GPR and ICU mortality after covariate adjustment. For both plots, hazard ratios (HRs) and 95% confidence intervals (CIs) are shown. The analysis includes subgroups based on age (≤70 years and >70 years), sex, BMI (<27.4 kg/m², 27.4–31.2 kg/m², ≥31.2 kg/m²), hypertension, type 2 diabetes, heart failure, CKD, stroke, AKI, CRRT, and mechanical ventilation. The P value for interaction is provided for each subgroup analysis.

The directional trends in the effect estimates for in-hospital mortality (**Figure 5A**) in almost subgroups were consistent with the overall outcomes before adjustment for covariates. Similarly, almost all subgroups were consistent with the overall outcome of ICU mortality (**Figure 5B**). In addition, there was an interaction between mechanical ventilation subgroup parameters (P < 0.01 for interaction). After adjustment for covariates, the directionality of the effect estimates in in-hospital and ICU mortality was consistent with the overall outcome in almost all subgroups except AKI and the subgroups with CRRT and no mechanical ventilation. There was no interaction between GPR and age, gender, BMI, hypertension, type 2 diabetes, heart failure, CKD, shock and mechanical ventilation (all P for interaction >0.05).

### 3.6 Sensitivity analyses

The E-values for the association between GPR and mortality outcomes at different time points are as follows: For ICU mortality, the E-values are 1.60 (30-day, HR=1.13), 1.79 (60-day, HR=1.23), and 1.85 (90-day, HR=1.26). For in-hospital mortality, the E-values are 1.11 (30-day, HR=1.05), 1.66 (60-day, HR=1.19), and 1.68 (90-day, HR=1.20). An E-value of 1.60 for 30-day ICU mortality implies that an unmeasured confounder would need to be associated with both the exposure and outcome by at least 1.60-fold to fully explain the observed association. Similarly, higher E-values for other time points indicate the minimum association strength required for potential unmeasured confounders to explain the observed results.

**Figure 6:**
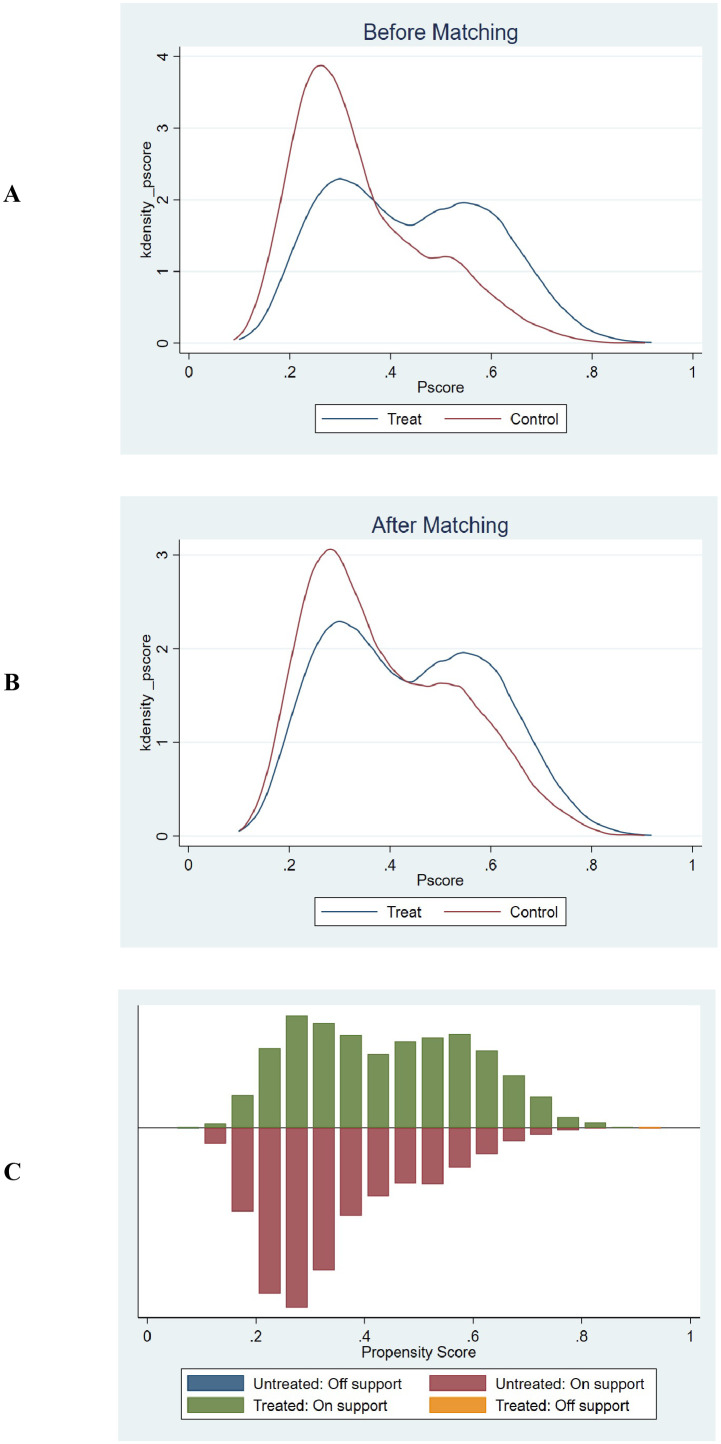
Propensity score matching and common support assessment regarding in-hospital mortality. **(A)** Kernel Density Estimation Before Matching: Displays the kernel density estimates of propensity scores for the treatment group (blue line) and control group (red line) prior to matching. The overlapping regions between the two curves indicate the initial common support area. Before matching, the density curves show some overlap, but there are also areas where the propensity scores of the treatment and control groups do not align closely, suggesting a limited common support region. **(B)** Kernel Density Estimation After Matching: Shows the kernel density estimates of propensity scores for the treatment group (blue line) and control group (red line) following matching. After matching, the density curves of the two groups are closely aligned across a wider range of propensity scores. This close alignment demonstrates an expanded common support region, indicating that the matching process has effectively balanced the distribution of propensity scores between the treatment and control groups. **(C)** Histogram of Common Support: Presents a histogram displaying the distribution of propensity scores for both the treatment and control groups. The green bars represent the treated observations within the common support range, the red bars represent the untreated observations within the common support range, the blue bar represents untreated observations outside the support, and the orange bar represents treated observations outside the support. The majority of observations fall within the common support range (indicated by the green and red bars), which means that only a minimal number of samples were excluded during the matching process. This ensures that the matched groups are highly comparable and reduces the potential for bias in the subsequent analysis.

**Figure 7:**
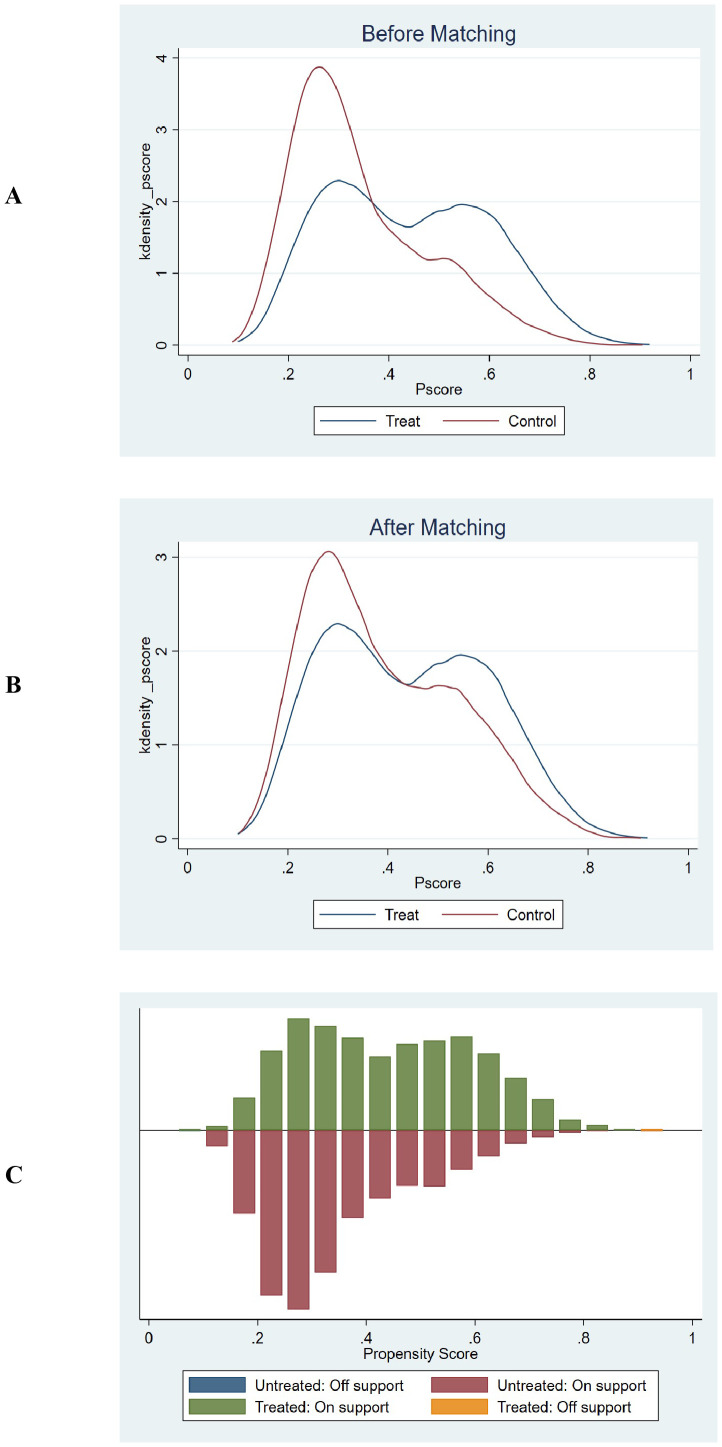
Propensity score matching and common support assessment regarding in-ICU mortality. **(A)** Kernel Density Estimation Before Matching: Displays the kernel density estimates of propensity scores for the treatment group (blue line) and control group (red line) prior to matching. The overlapping regions between the two curves indicate the initial common support area. Before matching, the density curves show some overlap, but there are also areas where the propensity scores of the treatment and control groups do not align closely, suggesting a limited common support region. **(B)** Kernel Density Estimation After Matching: Shows the kernel density estimates of propensity scores for the treatment group (blue line) and control group (red line) following matching. After matching, the density curves of the two groups are closely aligned across a wider range of propensity scores. This close alignment demonstrates an expanded common support region, indicating that the matching process has effectively balanced the distribution of propensity scores between the treatment and control groups. **(C)** Histogram of Common Support: Presents a histogram displaying the distribution of propensity scores for both the treatment and control groups. The green bars represent the treated observations within the common support range, the red bars represent the untreated observations within the common support range, the blue bar represents untreated observations outside the support, and the orange bar represents treated observations outside the support. The majority of observations fall within the common support range (indicated by the green and red bars), which means that only a minimal number of samples were excluded during the matching process. This ensures that the matched groups are highly comparable and reduces the potential for bias in the subsequent analysis.

The common support test results confirmed that the propensity scores of the high GPR and low GPR groups had sufficient overlap. The kernel density plots showed that the density lines for the two groups were closely aligned both before and after matching, indicating a large common support region. The histograms further demonstrated that most observations were within the common support range, ensuring minimal sample loss during the matching process. This confirmed the reliability of the matching process and the comparability of the matched groups. The balance assessment figures demonstrate that after PSM, the bias for all covariates was reduced to below 10%, and the t-tests showed no significant differences between the groups (p > 0.05). This indicates that the matching process successfully balanced the covariates between the high and low GPR groups. The kernel density and histogram figures show that the propensity scores of the two groups had sufficient overlap both before and after matching. After matching, the density lines and histogram bars for the two groups were closely aligned, indicating a large common support region and minimal loss of samples. This ensures that the matched groups are comparable and the results are reliable. These visualizations provide additional evidence of the effectiveness of the PSM method in reducing bias and enhancing the comparability of the groups, thereby strengthening the validity of the study findings. (**Supplementary Table 2**, **Figures 6**, **7**).

## 4 Discussion

This study examines the association between GPR and short- and long-term all-cause mortality in ICU-admitted sepsis patients using the MIMIC-IV database. With a large sample and extensive confounder adjustment, the results show a significant link between higher GPR and increased mortality risk in both hospital and ICU settings over 90 days. The nonlinear relationship identified by restricted cubic spline regression, with an inflection point at GPR 30, adds depth to GPR’s prognostic potential. Our study is the first large-scale validation of GPR in ICU sepsis patients, addressing inconsistencies in prior literature (13, 23). The composite GPR captures synergistic metabolic dysregulation (hyperglycemia + hypokalemia), explaining its incremental prognostic value over isolated markers. The U-shaped association—lower risk in Q2/Q3 vs. Q1—may reflect protective effects of moderate metabolic stress, whereas extremes (Q1: hypokalemia; Q4: severe dysregulation) drive mortality. The former is likely to exacerbate cardiac instability, while the latter’s extreme dysregulation overrides compensatory mechanisms. This aligns with the RCS-identified threshold (GPR=30), beyond which mortality risk escalates sharply. Sensitivity analyses including E-value quantification and propensity score matching further reinforced the robustness of our primary findings. The E-values (1.60–1.85 for ICU mortality) indicate that unmeasured confounders would need strong associations to nullify our results, while PSM confirmed the mortality gradient across quartiles in matched cohorts. These findings underscore GPR’s utility as a prognostic indicator in critically ill septic patients.

This study underscores that GPR, when evaluated both as a continuous variable and within categorized quartiles, stands out as a predictive marker for mortality in septic patients requiring intensive care. In particular, patients belonging to the highest GPR quartile (Q4) consistently demonstrated notably higher mortality rates compared to those in the lowest quartile (Q1) across all measured intervals (30, 60, and 90 days) and settings (hospital and ICU), as shown by Hazard Ratios (HRs) that reflected increased risk. These findings highlight the GPR’s potential as an independent prognostic indicator beyond traditional physiological and biochemical markers often used in ICU settings. While our study offers novel insights into the prognostic role of GPR in sepsis, it builds upon a modest body of prior research investigating GPR in various medical contexts. In non-septic conditions, such as myocardial infarction (7) and heart failure (6), elevated GPRs have also demonstrated correlations with increased morbidity and mortality, signifying its broad potential as a marker of metabolic imbalance. In ischemic stroke patients, a study (24) found that GPR was positively correlated with 30-day mortality, and the relationship between them was linear. In a multicenter retrospective cohort study (25), baseline GPR serum was found to be an independent predictor of all-cause mortality within 12 months in patients with acute and subacute ischemic stroke, and the study by Zhang et al. (26) also reached a similar conclusion. Chen et al. (27) found that high GPR was an independent risk factor for in-hospital mortality in patients with Acute type A aortic dissection (ATAAD). Serum GPR was observed in 146 patients. In cases of severe traumatic brain injury is substantially associated with trauma severity and 30-day mortality (28), and a similar association has been observed in patients with traumatic brain injury undergoing emergency craniotomy (29). Similarly, another study (30) observed a significant relationship between serum GPR and admission injury severity and the 6-month prognosis acute traumatic Spinal cord injurypatients. A high GFR correlated with Hunt and Kosnik grade and was also observed in patients with aneurysmal subarachnoid hemorrhage at admission Glasgow Outcome Scale score at discharge (31, 32). The predictive value between GPR and adverse clinical outcomes was also preliminarily verified in patients with acute intracerebral hemorrhage. In a retrospective study (33), it was observed that the predictive efficacy of GRF for the diagnosis of massive pulmonary embolism and non-massive pulmonary embolism in ICU patients was higher than that of D-dimer. However, another study based on ICU patients (34) found that the mortality of patients with isolated blunt abdominal trauma was highly correlated with GFR, and the sensitivity and specificity of GRF were both higher than 70%. Such studies provide a contextual backdrop where the dysregulation of glucose and potassium levels has been similarly implicated in adverse outcomes, suggesting a possible cross-pathophysiological utility of the GPR. However, existing literature on GPR specifically within sepsis is relatively scant, and the findings have been inconclusive due to significant methodological variances and population differences.

The GPR in sepsis reflects intricate metabolic dysregulations that accompany the systemic inflammatory response characteristic of this condition. Understanding the potential pathological mechanisms that lead to changes in both glucose and potassium levels can provide valuable insights into the prognostic value and clinical significance of GPR in sepsis. In sepsis, hyperglycemia is a frequent occurrence due to a combination of increased hepatic glucose production and impaired peripheral glucose utilization. Stress-induced hormonal responses (35), including the release of cortisol, catecholamines, glucagon, and pro-inflammatory cytokines (36), like tumor necrosis factor-alpha and interleukins, stimulate hepatic gluconeogenesis and glycogenolysis. This hypermetabolic state is compounded by insulin resistance, which limits glucose uptake by peripheral tissues, further elevating blood glucose levels (37). The pathological mechanism of hyperglycemia in sepsis can exacerbate the disease’s course through a variety of pathways. Elevated glucose levels contribute to oxidative stress by generating advanced glycation end products (AGEs) (38), which promote inflammation and tissue injury. Hyperglycemia also impairs neutrophil function (39), thereby weakening the host immune response and increasing susceptibility to infections. Furthermore, it is associated with endothelial dysfunction (40, 41), facilitating microvascular thrombosis and impaired tissue perfusion, which can deteriorate organ function. Clinically, the presence of hyperglycemia in sepsis patients has been linked to worse outcomes, including increased mortality rates, prolonged ICU stay, and higher incidences of multi-organ failure (42). This underlines the importance of close glycemic control in critical care settings, although the potential benefits must be weighed against the risks of hypoglycemia. Potassium imbalances, notably hypokalemia, are also common in sepsis and can stem from several factors. These include intracellular shifts of potassium driven by insulin administration (43) (used therapeutically to control hyperglycemia), beta-adrenergic stimulation, and metabolic alkalosis, as well as increased renal losses due to activation of the renin-angiotensin-aldosterone system and nephrotoxic effects of medications or the sepsis itself. Alternatively, hyperkalemia can occur, particularly in cases of acute kidney injury or significant cellular lysis (44). The clinical consequences of potassium imbalances are profound. Hypokalemia may lead to arrhythmias, muscle weakness, and respiratory failure, while hyperkalemia can precipitate potentially fatal cardiac arrhythmias (45). Potassium levels are critical for the function of cells, particularly in excitable tissues such as nerves and muscles, including the heart, implicating disturbances in significant morbidity in septic patients (46).

The ratio of serum glucose to potassium, or GPR, synthesizes the metabolic derangements of these two crucial solutes into a single metric. While each component on its own provides insight into specific pathophysiological processes, the GPR captures the overarching metabolic stress within the body (47). A high GPR may indicate a metabolic milieu marked by severe insulin resistance, profound stress response, and possibly inadequate compensatory mechanisms for electrolyte maintenance (48). This composite biomarker might therefore reflect a higher severity of systemic physiological derangement, correlating with worse clinical outcomes. Integrating glucose and potassium levels into a single ratio could afford a fuller picture of the metabolic state in sepsis compared to evaluating each element in isolation. In clinical practice, monitoring the GPR in sepsis patients could potentially aid in identifying patients at higher risk of adverse outcomes, offering opportunities for early intervention and more tailored therapeutic strategies. However, understanding the precise interplay and optimizing clinical use of GPR necessitate further research exploring the dynamic interrelations between glucose and potassium metabolisms in the progression of sepsis.

The E-values calculated for the association between GPR and mortality outcomes provide additional insight into the robustness of our findings against unmeasured confounding. For instance, an E-value of 1.60 for 30-day ICU mortality implies that an unmeasured confounder would need to be associated with both the exposure and outcome by at least 1.60-fold to fully explain the observed association. Similarly, the propensity score matching (PSM) analysis confirmed the consistency of our findings, further strengthening the validity of the observed association between GPR and mortality in sepsis patients.

This study’s contribution to the field is highlighted by its significant dataset derived from the MIMIC-IV database, encompassing a variety of demographic and clinical variables not previously analyzed in this combination. By confirming the prognostic relevance of GPR across a diverse ICU population, our findings suggest this biomarker could play a critical role in advancing sepsis management protocols, potentially guiding therapeutic decisions to mitigate mortality risks more effectively. Future research should focus on prospective validation of GPR thresholds and exploration of GPR dynamics over the course of sepsis to better understand its prognostic implications. By identifying patients at high risk of poor outcomes early in their treatment course, clinicians could tailor more aggressive monitoring and intervention strategies, which might include tighter glucose control, more frequent electrolyte assessments, or enhanced cardiovascular monitoring. Such an approach could lead to better resource allocation in high-intensity care environments and possibly improve patient outcomes by preemptively managing predicted complications.

This study also has limitations. The MIMIC-IV database consists largely of data from patients at a single tertiary care center, potentially limiting the generalizability of findings to other settings with different demographics, socioeconomic backgrounds, or healthcare systems (49). This can result in a population that is not fully representative of broader, more diverse sepsis populations worldwide. The demographic composition within the database may not sufficiently capture the variability across different ethnic and racial groups, which can affect disease presentation and responses to treatment, potentially skewing results and interpretations. Although the study includes adjustments for factors such as age and comorbidities, the inherent diversity in these variables may not be fully comparable across different demographic groups (50), implicating variations in baseline mortality risk that might confound the association between GPR and outcomes. In addition, as a retrospective study, it is subject to inherent biases such as selection bias and information bias (51). Decisions regarding data extraction and the variables included can introduce unintended biases that might impact the overall interpretation of findings. Despite efforts to adjust for numerous confounders, it is possible that not all relevant factors were considered or measured accurately, leading to residual confounding. Factors such as medication usage, nutritional status, or patient management differences might not be fully accounted for. The timing of GPR measurement relative to the onset of sepsis or the clinical course has not been standardized (52), potentially impacting its reliability as a consistent prognostic tool. The variation in when glucose and potassium levels are recorded can introduce discrepancies in how the GPR is calculated and interpreted. What’s more, a notable limitation is the potential for missing data, as not all patients may have fully recorded laboratory measurements or clinical outcomes. The study relied on multiple imputation methods to address missing data, which may introduce bias if assumptions about missingness are incorrect (53). The dataset may lack comprehensive longitudinal data necessary to explore causal relationships over time, limiting insights into how changes in GPR might reflect disease progression or response to interventions. Certain clinical variables crucial for understanding individual patient conditions, such as specific dietary intake, detailed medication histories, and underlying genetic predispositions (54, 55), may not be captured in the database, affecting the depth of analysis. Given the nature of the database as an aggregation of EMR from clinical practice, the quality and precision of recorded data can be variable. This variability may affect the accuracy of the input data, especially laboratory measurements, and the resulting analysis (56, 57). Notably, the lack of data on treatment interventions such as insulin therapy and fluid resuscitation represent a key limitation, as these factors can significantly influence patient outcomes and may confound the relationship between GPR and mortality (58, 59).

As a path forward, prospective studies evaluating GPR longitudinally across different stages of sepsis, and within broader and more varied populations, could validate our findings. Investigations might also focus on optimal intervention strategies for patients identified as high-risk by their GPR, possibly examining the impact of targeted therapies aimed at normalizing glucose and potassium homeostasis (60). Furthermore, establishing standardized GPR thresholds and developing clinical guidelines for their use could facilitate more widespread integration of GPR into ICU protocols. Limitations of our study, such as its retrospective nature and reliance on a single database, should also be addressed in future studies to enhance generalizability (61). Additionally, detailed longitudinal data collection could enable a better understanding of the causal pathways potentially involved in the links between GPR and sepsis outcomes.

## 5 Conclusion

In summary, the serum glucose-potassium ratio emerges from our investigation as a promising biomarker of mortality risk in sepsis, warranting further exploration and validation in future clinical research endeavors. By enhancing our understanding and application of GPR, healthcare practitioners may improve prognostic accuracy and patient outcomes in the challenging realm of sepsis management.

## Data Availability

The datasets presented in this study can be found in online repositories. The names of the repository/repositories and accession number(s) can be found in the article/**Supplementary Material**.
