# Supplementary material for: Association between serum glucose potassium ratio and short- and long-term all-cause mortality in patients with sepsis admitted to the intensive care unit: a retrospective analysis based on the MIMIC-IV database
Source: Front Endocrinol (Lausanne). 2025 Jul 30;16:1555082. doi: 10.3389/fendo.2025.1555082 (PMC12343221; doi:10.3389/fendo.2025.1555082)
Supplement: Supplementary file 1 [file Table1.docx]

| Supplementary Table 1. Summary of all characteristics of the study population. | | | | | | | |
| --- | --- | --- | --- | --- | --- | --- | --- |
| Variables | Total (n = 9,108) | Q1 (n = 2,272) | Q2 (n = 2,282) | Q3 (n = 2,277) | Q4 (n = 2,277) | Statistic | *P* |
|  |  |  |  |  |  |  |  |
| ***Characteristics*** |  |  |  |  |  |  |  |
| Age (year) | 71.61 ± 14.73 | 72.07 ± 14.94 | 71.52 ± 14.98 | 71.95 ± 14.52 | 70.91 ± 14.45 | F=2.91 | **0.033** |
| Weight (kg) | 79.16 ± 23.62 | 77.69 ± 23.65 | 77.85 ± 23.39 | 78.96 ± 22.97 | 82.15 ± 24.17 | F=17.45 | **<0.001** |
| Height (cm) | 168.44 ± 10.74 | 168.74 ± 10.88 | 168.58 ± 10.31 | 168.08 ± 10.94 | 168.38 ± 10.81 | F=0.88 | 0.448 |
| Gender (n(%)) |  |  |  |  |  | χ²=13.82 | **0.003** |
| F | 4038 (44.33) | 944 (41.55) | 993 (43.51) | 1046 (45.94) | 1055 (46.33) |  |  |
| M | 5070 (55.67) | 1328 (58.45) | 1289 (56.49) | 1231 (54.06) | 1222 (53.67) |  |  |
| Insurance, n(%) |  |  |  |  |  | χ²=8.91 | 0.179 |
| Medicaid | 578 (6.35) | 169 (7.44) | 143 (6.27) | 134 (5.88) | 132 (5.80) |  |  |
| Medicare | 5078 (55.75) | 1257 (55.33) | 1261 (55.26) | 1257 (55.20) | 1303 (57.22) |  |  |
| Other | 3452 (37.90) | 846 (37.24) | 878 (38.48) | 886 (38.91) | 842 (36.98) |  |  |
| Language, n(%) |  |  |  |  |  | χ²=2.16 | 0.540 |
| Other | 1038 (11.40) | 244 (10.74) | 259 (11.35) | 276 (12.12) | 259 (11.37) |  |  |
| English | 8070 (88.60) | 2028 (89.26) | 2023 (88.65) | 2001 (87.88) | 2018 (88.63) |  |  |
| Marital Status, n(%) |  |  |  |  |  | χ²=51.12 | **<0.001** |
| Divorced | 644 (7.07) | 165 (7.26) | 163 (7.14) | 168 (7.38) | 148 (6.50) |  |  |
| Married | 3801 (41.73) | 944 (41.55) | 960 (42.07) | 937 (41.15) | 960 (42.16) |  |  |
| NA | 1017 (11.17) | 200 (8.80) | 229 (10.04) | 264 (11.59) | 324 (14.23) |  |  |
| Single | 2127 (23.35) | 573 (25.22) | 564 (24.72) | 497 (21.83) | 493 (21.65) |  |  |
| Widowed | 1519 (16.68) | 390 (17.17) | 366 (16.04) | 411 (18.05) | 352 (15.46) |  |  |
| ***Laboratory parameters*** |  |  |  |  |  |  |  |
| WBC (×10^9^/L) | 13.76 ± 12.36 | 13.37 ± 13.52 | 13.08 ± 11.54 | 13.72 ± 9.73 | 14.88 ± 14.11 | F=9.27 | **<0.001** |
| RBC (×10^12^/L) | 3.42 ± 0.70 | 3.32 ± 0.68 | 3.38 ± 0.66 | 3.46 ± 0.71 | 3.51 ± 0.73 | F=35.03 | **<0.001** |
| Lymphocytes (×10^9^/L) | 1.70 ± 9.96 | 2.13 ± 15.86 | 1.83 ± 10.58 | 1.27 ± 3.41 | 1.62 ± 6.01 | F=0.54 | 0.657 |
| Platelet (×10^9^/L) | 197.82 ± 114.67 | 194.19 ± 123.05 | 198.91 ± 115.22 | 199.73 ± 113.48 | 198.41 ± 106.34 | F=1.05 | 0.368 |
| Hemoglobin (g/dL) | 10.23 ± 1.97 | 9.93 ± 1.90 | 10.15 ± 1.86 | 10.36 ± 2.01 | 10.47 ± 2.07 | F=34.01 | **<0.001** |
| RDW (%) | 16.00 ± 2.51 | 16.48 ± 2.63 | 15.96 ± 2.47 | 15.88 ± 2.50 | 15.67 ± 2.36 | F=43.44 | **<0.001** |
| Hematocrit (%) | 31.29 ± 5.92 | 30.60 ± 5.83 | 30.95 ± 5.52 | 31.60 ± 6.01 | 32.02 ± 6.19 | F=26.51 | **<0.001** |
| Albumin (g/L) | 2.91 ± 0.65 | 2.86 ± 0.65 | 2.89 ± 0.64 | 2.96 ± 0.67 | 2.93 ± 0.65 | F=4.76 | **0.003** |
| Sodium (mmol/L) | 138.56 ± 5.76 | 137.62 ± 5.44 | 138.56 ± 5.55 | 138.81 ± 5.43 | 139.24 ± 6.44 | F=32.59 | **<0.001** |
| Potassium (mmol/L) | 4.26 ± 0.64 | 4.60 ± 0.68 | 4.25 ± 0.58 | 4.14 ± 0.57 | 4.06 ± 0.59 | F=344.80 | **<0.001** |
| Total calcium (mmol/L) | 8.23 ± 0.83 | 8.25 ± 0.81 | 8.24 ± 0.80 | 8.22 ± 0.78 | 8.21 ± 0.92 | F=1.09 | 0.353 |
| Chlorine (mmol/L) | 104.06 ± 7.03 | 103.48 ± 6.87 | 104.42 ± 6.73 | 104.20 ± 6.74 | 104.12 ± 7.69 | F=7.51 | **<0.001** |
| Glucose (mmol/L) | 148.62 ± 64.05 | 96.47 ± 18.25 | 122.39 ± 17.48 | 148.66 ± 23.13 | 226.90 ± 75.54 | F=4205.85 | **<0.001** |
| Anion gap (mmol/L) | 15.73 ± 4.61 | 15.99 ± 4.95 | 14.97 ± 4.21 | 15.33 ± 4.21 | 16.65 ± 4.85 | F=60.36 | **<0.001** |
| pH | 7.35 ± 0.09 | 7.34 ± 0.09 | 7.36 ± 0.08 | 7.36 ± 0.08 | 7.35 ± 0.10 | F=32.84 | **<0.001** |
| PCO_2_ (mmHg) | 41.76 ± 11.39 | 42.69 ± 12.88 | 41.85 ± 10.96 | 41.51 ± 11.13 | 41.13 ± 10.57 | F=5.78 | **<0.001** |
| PO_2_ (mmHg) | 118.76 ± 71.09 | 113.39 ± 72.25 | 122.64 ± 73.70 | 121.03 ± 69.82 | 117.69 ± 68.65 | F=5.45 | **<0.001** |
| Free calcium (mmol/L) | 1.10 ± 0.11 | 1.11 ± 0.11 | 1.11 ± 0.10 | 1.10 ± 0.10 | 1.10 ± 0.11 | F=3.67 | **0.012** |
| PT (s) | 18.31 ± 10.24 | 19.64 ± 11.10 | 17.67 ± 8.72 | 17.64 ± 9.14 | 18.31 ± 11.56 | F=17.03 | **<0.001** |
| Fibrinogen (mg/dL) | 310.32 ± 187.88 | 287.49 ± 173.05 | 291.86 ± 171.25 | 335.99 ± 201.95 | 330.48 ± 201.03 | F=11.33 | **<0.001** |
| PPT (s) | 40.89 ± 20.28 | 40.87 ± 17.61 | 39.63 ± 18.68 | 40.39 ± 20.54 | 42.65 ± 23.60 | F=8.20 | **<0.001** |
| INR | 1.68 ± 0.99 | 1.82 ± 1.12 | 1.62 ± 0.86 | 1.63 ± 0.97 | 1.66 ± 1.00 | F=18.18 | **<0.001** |
| Total bilirubin (mg/dL) | 2.79 ± 5.88 | 3.67 ± 6.79 | 2.78 ± 5.87 | 2.84 ± 6.08 | 1.92 ± 4.47 | F=22.47 | **<0.001** |
| Direct bilirubin (mg/dL) | 4.24 ± 5.73 | 5.05 ± 6.18 | 4.33 ± 5.84 | 4.09 ± 5.52 | 3.29 ± 5.12 | F=2.88 | **0.035** |
| Indirect bilirubin (mg/dL) | 2.22 ± 2.85 | 2.65 ± 3.45 | 2.45 ± 2.83 | 1.85 ± 2.32 | 1.86 ± 2.46 | F=3.36 | **0.019** |
| ALT (U/L) | 167.94 ± 596.01 | 186.59 ± 784.00 | 134.50 ± 479.30 | 140.58 ± 452.44 | 205.54 ± 597.57 | F=5.00 | **0.002** |
| AST (U/L) | 302.91 ± 1065.00 | 349.66 ± 1235.43 | 253.35 ± 858.19 | 234.05 ± 758.68 | 366.35 ± 1273.72 | F=5.79 | **<0.001** |
| Urea nitrogen (mmol/L) | 35.33 ± 25.42 | 39.73 ± 27.33 | 32.31 ± 24.14 | 32.67 ± 23.03 | 36.64 ± 26.23 | F=44.41 | **<0.001** |
| Creatinine (mg/dL) | 1.77 ± 1.60 | 2.17 ± 1.96 | 1.57 ± 1.39 | 1.61 ± 1.47 | 1.74 ± 1.42 | F=70.06 | **<0.001** |
| LDH (U/L) | 715.76 ± 1616.90 | 828.65 ± 2105.51 | 633.76 ± 1211.17 | 563.17 ± 1040.05 | 829.50 ± 1828.93 | F=7.34 | **<0.001** |
| CK (U/L) | 1108.68 ± 6572.56 | 875.11 ± 5436.10 | 1036.51 ± 7800.22 | 1277.59 ± 7769.62 | 1203.38 ± 4870.74 | F=0.69 | 0.561 |
| CKMB (U/L) | 21.15 ± 52.72 | 14.24 ± 34.27 | 17.83 ± 48.56 | 21.76 ± 51.27 | 28.24 ± 65.76 | F=12.94 | **<0.001** |
| Troponint (μg/L) | 0.75 ± 2.38 | 0.39 ± 1.15 | 0.61 ± 1.71 | 0.71 ± 2.06 | 1.14 ± 3.43 | F=16.66 | **<0.001** |
| NT-proBNP (pmol/L) | 10065.50 ± 12234.37 | 11888.63 ± 12317.15 | 8313.65 ± 10569.28 | 9541.94 ± 12367.29 | 10538.55 ± 13343.72 | F=2.69 | **0.045** |
| ***Treatment*** |  |  |  |  |  |  |  |
| CRRT (n(%)) |  |  |  |  |  | χ²=18.04 | **<0.001** |
| No | 8297 (91.10) | 2026 (89.17) | 2112 (92.55) | 2091 (91.83) | 2068 (90.82) |  |  |
| Yes | 811 (8.90) | 246 (10.83) | 170 (7.45) | 186 (8.17) | 209 (9.18) |  |  |
| CRRT (days) | 5.87 ± 6.35 | 5.71 ± 6.75 | 6.59 ± 7.25 | 5.80 ± 5.68 | 5.53 ± 5.60 | F=0.99 | 0.399 |
| Ventilation (n(%)) |  |  |  |  |  | χ²=7.36 | 0.061 |
| No | 1459 (16.02) | 398 (17.52) | 358 (15.69) | 333 (14.62) | 370 (16.25) |  |  |
| Yes | 7649 (83.98) | 1874 (82.48) | 1924 (84.31) | 1944 (85.38) | 1907 (83.75) |  |  |
| Ventilation (hours) | 101.88 ± 145.10 | 91.36 ± 144.60 | 99.46 ± 141.63 | 108.83 ± 152.51 | 107.59 ± 140.66 | F=5.94 | **<0.001** |
| ***Comorbidity*** |  |  |  |  |  |  |  |
| Hypertension (n(%)) |  |  |  |  |  | χ²=41.33 | **<0.001** |
| No | 5615 (61.65) | 1526 (67.17) | 1394 (61.09) | 1350 (59.29) | 1345 (59.07) |  |  |
| Yes | 3493 (38.35) | 746 (32.83) | 888 (38.91) | 927 (40.71) | 932 (40.93) |  |  |
| Type 2 diabetes mellitus (n(%)) |  |  |  |  |  | χ²=640.05 | **<0.001** |
| No | 6235 (68.46) | 1760 (77.46) | 1787 (78.31) | 1599 (70.22) | 1089 (47.83) |  |  |
| Yes | 2873 (31.54) | 512 (22.54) | 495 (21.69) | 678 (29.78) | 1188 (52.17) |  |  |
| Heart failure (n(%)) |  |  |  |  |  | χ²=5.36 | 0.147 |
| No | 5905 (64.83) | 1463 (64.39) | 1509 (66.13) | 1495 (65.66) | 1438 (63.15) |  |  |
| Yes | 3203 (35.17) | 809 (35.61) | 773 (33.87) | 782 (34.34) | 839 (36.85) |  |  |
| Myocardial infarct (n(%)) |  |  |  |  |  | χ²=50.89 | **<0.001** |
| No | 8397 (92.19) | 2139 (94.15) | 2130 (93.34) | 2104 (92.40) | 2024 (88.89) |  |  |
| Yes | 711 (7.81) | 133 (5.85) | 152 (6.66) | 173 (7.60) | 253 (11.11) |  |  |
| Malignant tumor (n(%)) |  |  |  |  |  | χ²=35.32 | **<0.001** |
| No | 7061 (77.53) | 1719 (75.66) | 1720 (75.37) | 1758 (77.21) | 1864 (81.86) |  |  |
| Yes | 2047 (22.47) | 553 (24.34) | 562 (24.63) | 519 (22.79) | 413 (18.14) |  |  |
| Chronic kidney diseases (n(%)) |  |  |  |  |  | χ²=17.68 | **<0.001** |
| No | 6884 (75.58) | 1663 (73.20) | 1776 (77.83) | 1753 (76.99) | 1692 (74.31) |  |  |
| Yes | 2224 (24.42) | 609 (26.80) | 506 (22.17) | 524 (23.01) | 585 (25.69) |  |  |
| Acute renal failure (n(%)) |  |  |  |  |  | χ²=27.54 | **<0.001** |
| No | 4374 (48.02) | 1011 (44.50) | 1188 (52.06) | 1106 (48.57) | 1069 (46.95) |  |  |
| Yes | 4734 (51.98) | 1261 (55.50) | 1094 (47.94) | 1171 (51.43) | 1208 (53.05) |  |  |
| Cirrhosis (n(%)) |  |  |  |  |  | χ²=42.28 | **<0.001** |
| No | 7998 (87.81) | 1934 (85.12) | 1979 (86.72) | 2009 (88.23) | 2076 (91.17) |  |  |
| Yes | 1110 (12.19) | 338 (14.88) | 303 (13.28) | 268 (11.77) | 201 (8.83) |  |  |
| Hepatitis (n(%)) |  |  |  |  |  | χ²=7.06 | 0.070 |
| No | 8669 (95.18) | 2147 (94.50) | 2162 (94.74) | 2174 (95.48) | 2186 (96.00) |  |  |
| Yes | 439 (4.82) | 125 (5.50) | 120 (5.26) | 103 (4.52) | 91 (4.00) |  |  |
| Tuberculosis (n(%)) |  |  |  |  |  | χ²=3.35 | 0.341 |
| No | 8776 (96.35) | 2198 (96.74) | 2186 (95.79) | 2199 (96.57) | 2193 (96.31) |  |  |
| Yes | 332 (3.65) | 74 (3.26) | 96 (4.21) | 78 (3.43) | 84 (3.69) |  |  |
| Pneumonia (n(%)) |  |  |  |  |  | χ²=5.62 | 0.132 |
| No | 5211 (57.21) | 1343 (59.11) | 1312 (57.49) | 1277 (56.08) | 1279 (56.17) |  |  |
| Yes | 3897 (42.79) | 929 (40.89) | 970 (42.51) | 1000 (43.92) | 998 (43.83) |  |  |
| Stroke (n(%)) |  |  |  |  |  | χ²=25.50 | **<0.001** |
| No | 8138 (89.35) | 2068 (91.02) | 2054 (90.01) | 2043 (89.72) | 1973 (86.65) |  |  |
| Yes | 970 (10.65) | 204 (8.98) | 228 (9.99) | 234 (10.28) | 304 (13.35) |  |  |
| Hyperlipidemia, (n(%)) |  |  |  |  |  | χ²=37.19 | **<0.001** |
| No | 6215 (68.24) | 1628 (71.65) | 1572 (68.89) | 1570 (68.95) | 1445 (63.46) |  |  |
| Yes | 2893 (31.76) | 644 (28.35) | 710 (31.11) | 707 (31.05) | 832 (36.54) |  |  |
| COPD (n(%)) |  |  |  |  |  | χ²=3.10 | 0.376 |
| No | 8260 (90.69) | 2080 (91.55) | 2068 (90.62) | 2051 (90.07) | 2061 (90.51) |  |  |
| Yes | 848 (9.31) | 192 (8.45) | 214 (9.38) | 226 (9.93) | 216 (9.49) |  |  |
| Acute kidney injury stage (n(%)) |  |  |  |  |  | χ²=34.38 | **<0.001** |
| 1 | 1403 (18.94) | 356 (19.63) | 347 (18.89) | 353 (18.76) | 347 (18.51) |  |  |
| 2 | 3087 (41.67) | 666 (36.71) | 835 (45.45) | 810 (43.04) | 776 (41.39) |  |  |
| 3 | 2918 (39.39) | 792 (43.66) | 655 (35.66) | 719 (38.20) | 752 (40.11) |  |  |
| ***Scoring systems*** |  |  |  |  |  |  |  |
| SOFA score (score) | 6.77 ± 3.90 | 7.15 ± 4.08 | 6.23 ± 3.65 | 6.52 ± 3.74 | 7.18 ± 4.02 | F=33.60 | **<0.001** |
| APSIII score (score) | 58.48 ± 23.59 | 60.89 ± 24.31 | 53.75 ± 21.65 | 56.23 ± 21.93 | 63.08 ± 25.13 | F=76.14 | **<0.001** |
| SAPSII score (score) | 45.83 ± 14.75 | 47.50 ± 15.57 | 43.74 ± 13.71 | 44.94 ± 14.03 | 47.14 ± 15.28 | F=34.07 | **<0.001** |
| OASIS, score (score) | 36.09 ± 8.90 | 36.05 ± 8.95 | 35.07 ± 8.57 | 35.91 ± 8.70 | 37.32 ± 9.24 | F=24.98 | **<0.001** |
| GCS score (score) | 13.06 ± 3.18 | 13.12 ± 3.08 | 13.23 ± 2.90 | 13.07 ± 3.15 | 12.84 ± 3.53 | F=6.07 | **<0.001** |
| Charlson score (score) | 6.53 ± 2.81 | 6.69 ± 2.81 | 6.42 ± 2.76 | 6.42 ± 2.79 | 6.59 ± 2.87 | F=5.23 | **<0.001** |
| SIRS score (score) |  |  |  |  |  | χ²=110.42 | **<0.001** |
| 0 | 60 (0.66) | 18 (0.79) | 19 (0.83) | 13 (0.57) | 10 (0.44) |  |  |
| 1 | 637 (6.99) | 188 (8.27) | 187 (8.19) | 145 (6.37) | 117 (5.14) |  |  |
| 2 | 2302 (25.27) | 655 (28.83) | 629 (27.56) | 549 (24.11) | 469 (20.60) |  |  |
| 3 | 3837 (42.13) | 916 (40.32) | 939 (41.15) | 993 (43.61) | 989 (43.43) |  |  |
| 4 | 2272 (24.95) | 495 (21.79) | 508 (22.26) | 577 (25.34) | 692 (30.39) |  |  |
| Continuous variables are expressed as the median and interquartile range. Counting data are presented as numbers and percentages. The medical condition was defined based on the ICD-9 code. WBC, white blood cell; RBC, red blood cell; RDW, red blood cell distribution width; PCO_2_, partial pressure of carbon dioxide; PO_2_, partial pressure of oxygen; LD, Lactate Dehydrogenase; PT, prothrombin time; PTT, partial thromboplastin time; INR, international normalized ratio; ALT, alanine aminotransferase; AST, aspartate aminotransferas; CKMB, [creatine kinase-MB](https://www.baidu.com/s?sa=re_dqa_generate&wd=Creatine%20Kinase-MB&rsv_pq=9e8969a8000ac7a0&oq=BLD%20(mg/L)%20BCK%20(mmol/L)%20CKMB%20(U/L)%E8%8B%B1%E6%96%87%E5%85%A8%E7%A7%B0%E6%98%AF%E4%BB%80%E4%B9%88&rsv_t=66cdhrKelPNe7ps1jBwvXowxl99uKKHgyKn4cwdxJVl0a2Rf3E32xRksUnk&tn=baidu&ie=utf-8); BCK, [blood ketone](https://www.baidu.com/s?sa=re_dqa_generate&wd=Blood%20Ketone&rsv_pq=9e8969a8000ac7a0&oq=BLD%20(mg/L)%20BCK%20(mmol/L)%20CKMB%20(U/L)%E8%8B%B1%E6%96%87%E5%85%A8%E7%A7%B0%E6%98%AF%E4%BB%80%E4%B9%88&rsv_t=66cdhrKelPNe7ps1jBwvXowxl99uKKHgyKn4cwdxJVl0a2Rf3E32xRksUnk&tn=baidu&ie=utf-8); NT-proBNP, N-terminal pro-brain natriuretic peptide; CRRT, continuous renal replacement therapy; COPD, chronic obstructive pulmonary disease; OASIS, oxford acute severity of illness score; SASPII, simplified acute physiology score II; SOFA, sequential organ failure assessment; CNS, central nervous system; GCS, Glasgow Coma Scale; SIRS, Systemic Inflammatory Response Syndrome; F, ANOVA; χ², Chi-square test; SD, standard deviation | | | | | | | |
