# Supplementary material for: Association between serum glucose potassium ratio and short- and long-term all-cause mortality in patients with sepsis admitted to the intensive care unit: a retrospective analysis based on the MIMIC-IV database
Source: Front Endocrinol (Lausanne). 2025 Jul 30;16:1555082. doi: 10.3389/fendo.2025.1555082 (PMC12343221; doi:10.3389/fendo.2025.1555082)
Supplement: Supplementary file 2 [file Table2.docx]

| Supplementary Table 2. Balance of Covariates Before and After Propensity Score Matching Regarding Mortality. | | | | | | | | |
| --- | --- | --- | --- | --- | --- | --- | --- | --- |
| Unatched |  | Mean |  |  | %reduct | t-test |  | V(T)/ |
| Variable | Matched | Treated | Control | %bias | bias | t | p>t | V(C) |
|  |  |  |  |  |  |  |  |  |
| Age | U | 1.5766 | 1.5988 | -4.5 |  | -2.09 | 0.037 | 1.02 |
|  | M | 1.5765 | 1.5814 | -1 | 77.8 | -0.41 | 0.679 | 1 |
|  |  |  |  |  |  |  |  |  |
| Gender | U | 0.53837 | 0.56782 | -5.9 |  | -2.75 | 0.006 | . |
|  | M | 0.53824 | 0.53809 | 0 | 99.5 | 0.01 | 0.99 | . |
|  |  |  |  |  |  |  |  |  |
| Race (unknown) | U | 0.23805 | 0.19381 | 10.8 |  | 5.03 | 0 | . |
|  | M | 0.23812 | 0.23899 | -0.2 | 98 | -0.08 | 0.933 | . |
|  |  |  |  |  |  |  |  |  |
| Race Asian | U | 0.03446 | 0.02953 | 2.8 |  | 1.31 | 0.19 | . |
|  | M | 0.03447 | 0.03636 | -1.1 | 61.8 | -0.42 | 0.672 | . |
|  |  |  |  |  |  |  |  |  |
| Race White | U | 0.6351 | 0.68117 | -9.7 |  | -4.52 | 0 | . |
|  | M | 0.63499 | 0.62992 | 1.1 | 89 | 0.44 | 0.662 | . |
|  |  |  |  |  |  |  |  |  |
| Race | U | 0.09065 | 0.09337 | -0.9 |  | -0.44 | 0.663 | . |
|  | M | 0.09067 | 0.09284 | -0.8 | 20.2 | -0.31 | 0.755 | . |
|  |  |  |  |  |  |  |  |  |
| BMI | U | 21.297 | 30.993 | -1.9 |  | -0.88 | 0.381 | 0.68* |
|  | M | 21.303 | 24.197 | -0.6 | 70.2 | -0.26 | 0.796 | 0.88* |
|  |  |  |  |  |  |  |  |  |
| Insurance (None) | U | 0.0585 | 0.06649 | -3.3 |  | -1.52 | 0.129 | . |
|  | M | 0.05852 | 0.06025 | -0.7 | 78.2 | -0.31 | 0.76 | . |
|  |  |  |  |  |  |  |  |  |
| Insurance Medicare | U | 0.55749 | 0.55756 | 0 |  | -0.01 | 0.995 | . |
|  | M | 0.55736 | 0.55895 | -0.3 | -2069.6 | -0.13 | 0.894 | . |
|  |  |  |  |  |  |  |  |  |
| Divorced | U | 0.0669 | 0.07303 | -2.4 |  | -1.11 | 0.268 | . |
|  | M | 0.06692 | 0.06489 | 0.8 | 66.9 | 0.34 | 0.734 | . |
|  |  |  |  |  |  |  |  |  |
| Married | U | 0.41819 | 0.4168 | 0.3 |  | 0.13 | 0.896 | . |
|  | M | 0.41831 | 0.42439 | -1.2 | -338.4 | -0.51 | 0.609 | . |
|  |  |  |  |  |  |  |  |  |
| Single | U | 0.21952 | 0.24209 | -5.4 |  | -2.47 | 0.014 | . |
|  | M | 0.21958 | 0.21756 | 0.5 | 91 | 0.2 | 0.839 | . |
|  |  |  |  |  |  |  |  |  |
| Wido | U | 0.16305 | 0.16905 | -1.6 |  | -0.75 | 0.456 | . |
|  | M | 0.1628 | 0.16773 | -1.3 | 18 | -0.55 | 0.582 | . |
|  |  |  |  |  |  |  |  |  |
| Hypertension | U | 0.40834 | 0.36835 | 8.2 |  | 3.81 | 0 | . |
|  | M | 0.40846 | 0.40629 | 0.4 | 94.6 | 0.18 | 0.854 | . |
|  |  |  |  |  |  |  |  |  |
| Type 2 Diabetes | U | 0.45931 | 0.22759 | 50.3 |  | 23.79 | 0 | . |
|  | M | 0.45944 | 0.46147 | -0.4 | 99.1 | -0.17 | 0.866 | . |
|  |  |  |  |  |  |  |  |  |
| Heart failure | U | 0.35853 | 0.34748 | 2.3 |  | 1.07 | 0.284 | . |
|  | M | 0.35863 | 0.35892 | -0.1 | 97.4 | -0.03 | 0.98 | . |
|  |  |  |  |  |  |  |  |  |
| MI | U | 0.10107 | 0.06401 | 13.5 |  | 6.41 | 0 | . |
|  | M | 0.1011 | 0.10211 | -0.4 | 97.3 | -0.14 | 0.889 | . |
|  |  |  |  |  |  |  |  |  |
| MT | U | 0.2007 | 0.23943 | -9.4 |  | -4.3 | 0 | . |
|  | M | 0.20075 | 0.20119 | -0.1 | 98.9 | -0.05 | 0.964 | . |
|  |  |  |  |  |  |  |  |  |
| CKD | U | 0.24674 | 0.24262 | 1 |  | 0.44 | 0.657 | . |
|  | M | 0.24681 | 0.24812 | -0.3 | 68.4 | -0.13 | 0.9 | . |
|  |  |  |  |  |  |  |  |  |
| Arf | U | 0.53229 | 0.51211 | 4 |  | 1.87 | 0.061 | . |
|  | M | 0.53216 | 0.51115 | 4.2 | -4.1 | 1.75 | 0.081 | . |
|  |  |  |  |  |  |  |  |  |
| Cirrhosis | U | 0.10078 | 0.13475 | -10.6 |  | -4.81 | 0 | . |
|  | M | 0.10081 | 0.10458 | -1.2 | 88.9 | -0.52 | 0.606 | . |
|  |  |  |  |  |  |  |  |  |
| Hepatitis | U | 0.0417 | 0.05217 | -4.9 |  | -2.26 | 0.024 | . |
|  | M | 0.04171 | 0.03882 | 1.4 | 72.3 | 0.61 | 0.54 | . |
|  |  |  |  |  |  |  |  |  |
| TB | U | 0.03562 | 0.03696 | -0.7 |  | -0.33 | 0.741 | . |
|  | M | 0.03563 | 0.03317 | 1.3 | -84.1 | 0.56 | 0.575 | . |
|  |  |  |  |  |  |  |  |  |
| Pneuonia | U | 0.43672 | 0.42246 | 2.9 |  | 1.33 | 0.182 | . |
|  | M | 0.43685 | 0.43091 | 1.2 | 58.4 | 0.5 | 0.619 | . |
|  |  |  |  |  |  |  |  |  |
| Stroke | U | 0.12366 | 0.09602 | 8.8 |  | 4.15 | 0 | . |
|  | M | 0.1237 | 0.13181 | -2.6 | 70.7 | -1.01 | 0.313 | . |
|  |  |  |  |  |  |  |  |  |
| HLP | U | 0.34926 | 0.29832 | 10.9 |  | 5.07 | 0 | . |
|  | M | 0.34907 | 0.35183 | -0.6 | 94.6 | -0.24 | 0.811 | . |
|  |  |  |  |  |  |  |  |  |
| COPD | U | 0.09731 | 0.09054 | 2.3 |  | 1.08 | 0.281 | . |
|  | M | 0.09733 | 0.08792 | 3.2 | -39.1 | 1.35 | 0.177 | . |
|  |  |  |  |  |  |  |  |  |
| WBC | U | 14.51 | 13.189 | 10.6 |  | 4.95 | 0 | 1.09* |
|  | M | 14.403 | 14.715 | -2.5 | 76.4 | -0.91 | 0.365 | 0.42* |
|  |  |  |  |  |  |  |  |  |
| RBC | U | 3.4797 | 3.3485 | 17.7 |  | 8.22 | 0 | 1.10* |
|  | M | 3.4798 | 3.4486 | 4.2 | 76.2 | 1.72 | 0.086 | 1.03 |
|  |  |  |  |  |  |  |  |  |
| RDW | U | 15.684 | 16.034 | -12.7 |  | -5.83 | 0 | 0.83* |
|  | M | 15.682 | 15.708 | -0.9 | 92.6 | -0.4 | 0.69 | 0.90* |
|  |  |  |  |  |  |  |  |  |
| ALT | U | 125.11 | 96.627 | 5.9 |  | 2.73 | 0.006 | 0.90* |
|  | M | 125.12 | 130.05 | -1 | 82.7 | -0.4 | 0.689 | 0.72* |
|  |  |  |  |  |  |  |  |  |
| AST | U | 223.74 | 177.06 | 5.3 |  | 2.5 | 0.013 | 1.48* |
|  | M | 223.76 | 233.96 | -1.1 | 78.2 | -0.46 | 0.649 | 1.19* |
|  |  |  |  |  |  |  |  |  |
| SOFA | U | 7.0145 | 6.6202 | 10.1 |  | 4.69 | 0 | 1.04 |
|  | M | 7.0113 | 7.1335 | -3.1 | 69 | -1.24 | 0.215 | 0.86* |
|  |  |  |  |  |  |  |  |  |
| APSiii | U | 61.119 | 56.869 | 18 |  | 8.38 | 0 | 1.11* |
|  | M | 61.085 | 61.767 | -2.9 | 84 | -1.13 | 0.26 | 0.86* |
|  |  |  |  |  |  |  |  |  |
| SAPSii | U | 46.509 | 45.416 | 7.4 |  | 3.43 | 0.001 | 1.04 |
|  | M | 46.491 | 46.948 | -3.1 | 58.1 | -1.23 | 0.218 | 0.87* |
|  |  |  |  |  |  |  |  |  |
| OASIS | U | 36.915 | 35.583 | 14.9 |  | 6.94 | 0 | 1.10* |
|  | M | 36.907 | 37.024 | -1.3 | 91.2 | -0.53 | 0.599 | 0.96 |
|  |  |  |  |  |  |  |  |  |
| GCS | U | 12.853 | 13.141 | -8.8 |  | -4.12 | 0 | 1.30* |
|  | M | 12.856 | 12.803 | 1.6 | 81.7 | 0.63 | 0.528 | 1.02 |

The table presents the balance of covariates before and after propensity score matching (PSM) for hospital mortality. Unmatched (U): Statistics for the unmatched sample, reflecting the original differences between the treatment (high GPR) and control (low GPR) groups. Matched (M): Statistics after PSM, showing the balanced state of covariates between the two groups. Mean: Mean values of the covariates for the treatment and control groups. %bias: Percentage bias of the covariates before matching, quantifying the imbalance between groups. %reduct: Percentage reduction in bias after matching, indicating the effectiveness of PSM in balancing covariates. t-test: t-test statistic for assessing differences in covariates between groups after matching. p>t: p-value from the t-test, with values >0.05 indicating no significant difference between groups (successful matching). V(T)/V(C): Variance ratio of the treatment to control group, with values within [0.94; 1.07] suggesting homogeneity of variances. Variables with a variance ratio outside the [0.94; 1.07] range are marked with an asterisk (*). Categorical variables were dummy-coded (e.g., divorced=1 vs. others=0). The successful PSM is evidenced by most covariates achieving balanced with %bias <10%, non-significant t-test p-values (>0.05), and appropriate variance ratios, ensuring reliable subseque.
